# Breast cancer metastasis: immune profiling of lymph nodes reveals exhaustion of effector T cells and immunosuppression

**DOI:** 10.1002/1878-0261.13047

**Published:** 2021-07-12

**Authors:** Inga Hansine Rye, Kanutte Huse, Sarah E. Josefsson, Wanja Kildal, Håvard E. Danielsen, Ellen Schlichting, Øystein Garred, Margit L. Riis, OSBREAC, Ole Christian Lingjærde, June H. Myklebust, Hege G. Russnes

**Affiliations:** ^1^ Department of Cancer Genetics Institute for Cancer Research Division of Cancer Medicine Oslo University Hospital Radiumhospitalet Oslo Norway; ^2^ Department of Cancer Immunology Institute for Cancer Research Division of Cancer Medicine Oslo University Hospital Radiumhospitalet Norway; ^3^ KG Jebsen Centre for B‐Cell Malignancies Institute for Clinical Medicine University of Oslo Norway; ^4^ Division of Cancer Medicine Institute for Cancer Genetics and Informatics Oslo University Hospital Radiumhospitalet Oslo Norway; ^5^ Department of Informatics University of Oslo Norway; ^6^ Nuffield Division of Clinical Laboratory Sciences University of Oxford UK; ^7^ Department of Oncology Division of Cancer Medicine Oslo University Hospital Norway; ^8^ Department of Pathology Division of Laboratory Medicine Oslo University Hospital Norway; ^9^ Oslo Breast Cancer Consortium Oslo University Hospital Norway; ^10^ Institute for Bioinformatics University of Oslo Norway

**Keywords:** breast cancer, immune activation, immune profile, metastatic lymph nodes, T‐cell exhaustion

## Abstract

Sentinel lymph nodes are the first nodes draining the lymph from a breast and could reveal early changes in the host immune system upon dissemination of breast cancer cells. To investigate this, we performed single‐cell immune profiling of lymph nodes with and without metastatic cells. Whereas no significant changes were observed for B‐cell and natural killer (NK)‐cell subsets, metastatic lymph nodes had a significantly increased frequency of CD8 T cells and a skewing toward an effector/memory phenotype of CD4 and CD8 T cells, suggesting an ongoing immune response. Additionally, metastatic lymph nodes had an increased frequency of TIGIT (T‐cell immunoreceptor with Ig and ITIM domains)‐positive T cells with suppressed TCR signaling compared with non‐metastatic nodes, indicating exhaustion of effector T cells, and an increased frequency of regulatory T cells (Tregs) with an activated phenotype. T‐cell alterations correlated with the percentage of metastatic tumor cells, reflecting the presence of metastatic tumor cells driving T effector cells toward exhaustion and promoting immunosuppression by recruitment or increased differentiation toward Tregs. These results show that immune suppression occurs already in early stages of tumor progression.

## Graphical abstract

Sentinel lymph nodes (SLN) are the first nodes draining the lymph from a breast and could reveal early changes in the host immune system upon dissemination of breast cancer (BC) cells. We used single‐cell mass cytometry and functional analyses of metastatic and non‐metastatic LN and revealed a dysfunctional and suppressed immune response. Metastatic LN had an increased frequency of TIGIT‐positive T cells with suppressed TCR signaling compared with non‐metastatic nodes and an increased frequency of Tregs with an activated phenotype. The alterations were correlated with metastatic tumor burden in the LN of patients with BC.

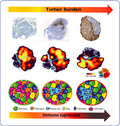

**Abbreviations**
LN: lymph nodes
SN: sentinel node
TIGIT: T‐cell immunoreceptor with Ig and ITIM domains
Tregs: regulatory T cells

## 1 Introduction: immune response in breast cancer patients

Breast cancer is the most common cancer and the second most common cause of cancer‐related death among women worldwide [1]. Breast cancer can be divided into different subtypes, each with a unique molecular profile, guided treatment and prognosis [2, 3]. Despite improved classification, a fundament for treatment decision is still the expression or absence of estrogen receptor (ER), progesterone receptor (PgR), human epidermal growth factor 2 receptor (HER2), and the proliferation marker Ki‐67. Breast cancer has commonly been considered immunogenic “cold,” since most tumors have low lymphocyte infiltration. However, recent studies have demonstrated that HER2+ and triple‐negative (ER‐, PgR‐, and HER2‐) tumors frequently have microenvironments infiltrated with immune cells. Within these groups, patients with high immune cell infiltration have improved recurrence‐free survival compared to patients with low immune cell infiltration [4, 5].

Detection of lymph node metastases is important for appropriate staging of the patient’s disease and closely linked to prognosis [6]. For patients with resectable breast cancer, diagnostic procedures include evaluation of regional lymph nodes (axilla), and if there is no sign of metastases, a sentinel node procedure will be performed. This reduces the morbidity associated with removal of all axillary lymph nodes [7, 8]. By definition, the sentinel lymph node is the first node draining the lymphatic from the tumor and thus represents a unique connection between the tumor and the host immune system. Approximately 30–40% of breast cancer patients have metastases in sentinel lymph nodes at time of diagnosis, and the size of the metastases is typically much smaller in sentinel than axillary lymph nodes [9]. Metastases in sentinel and axillary lymph nodes have been categorized into three groups based on size of the tumor deposits: macro‐metastasis (> 2 mm), micro‐metastasis (0.2–2 mm), and isolated tumor cells (< 0.2 mm) [6]. The clinical significance has been debated, but isolated tumor cells in axillary lymph nodes seem to have no impact on survival, whereas patients with micro‐metastasis demonstrate worse prognosis than node‐negative patients, and patients with macro‐metastasis in the axillary lymph node face the worst prognosis [10, 11].

The lymph nodes are the main repositories for B and T cells and serve as a meeting point where antigen‐presenting cells (APC) such as dendritic cells, macrophages, and B cells present antigens to T cells and initiate an adaptive immune response [12]. Tumor cells can present neoantigens via MHC class I directly to CD8 T cells, although efficient priming of naïve CD8 T cells to initiate an immune response requires antigen presentation by dendritic cells [13]. Most studies of immune response in breast cancer patients have focused on characterizing the primary tumor [14, 15], including recent studies using mass cytometry and imaging mass cytometry to comprehensively map tumor and immune cell ecosystems [16, 17]. These studies showed that the microenvironment plays crucial roles in promotion and suppression of tumor growth. Earlier studies of sentinel lymph nodes, based on PCR or immunohistochemical (IHC) analysis, identified increased gene expression level of *FOXP3*, indicative of increased level of regulatory T cells (Tregs), and reduced frequency of CD4 T cells and mature dendritic cells in nodes with metastasis as compared to nodes without tumor cells [18, 19, 20]. A separate study using multiparameter flow cytometry recently confirmed higher frequency of Tregs in tumor cell containing sentinel nodes [21]. However, the impact of metastatic tumor cells in lymph nodes on the immune cell composition and activation status has not been explored in detail. To identify changes in immune profiles upon metastasis, we applied mass cytometry single‐cell characterization of tumor and immune cells from metastatic and non‐metastatic lymph nodes in a cohort of 52 breast cancer patients.

## 2 Material and methods

### 2.1 Sample collection

The study was approved by the regional committee for research ethics (200606181‐1, 538‐07278a, 2009/4935, 2016/433). All samples were collected as part of the Oslo2 breast cancer observational trial with written patient consent in accordance with the Declaration of Helsinki. Sentinel and axillary lymph nodes from 52 breast cancer patients included in the study represented cases with primary operable breast cancer (cancer tumor stage (cT)1‐cT2) without distant metastases at time of diagnosis (Table S1). The sentinel lymph nodes were removed at time of primary surgery and prior to adjuvant therapy. The sentinel lymph nodes were categorized as metastatic (SNmet, *n* = 16) or non‐metastatic (SNneg, *n* = 18), whereas axillary lymph nodes were categorized as metastatic (ALNmet, *n* = 18) by two pathologists (ØG and HR). Reexamination revealed that four of the 18 ALNmet were probably not sampled from representative metastatic lymph nodes, but from non‐metastatic neighboring lymph nodes in the axilla. Morphology including histological grade and expression of standard clinical markers (ER, PgR, HER2, and Ki‐67) for the primary tumor were assessed according to current ASCO/CAP recommendations [22] (Table S1). The standard surgical procedure for sentinel node detection in Norway is subcutaneously periareolarly injection of radioactivity (20 MBq ^99m^ Tc‐Nanocoll (Tc99)), and blue dye and the one or two lymph nodes capturing the ink and/or the radioactivity first are regarded the sentinel node. After surgical removal, half of the node was sent to routine histopathology and examined while the other half was dissociated into single cells and cryopreserved in liquid nitrogen.

### 2.2 Dissociation protocol

The ALNmet samples were minced into small pieces and digested with 2 mg·mL^−1^ collagenase type IV (C5138, Sigma‐Aldrich, KgaA Merck, Darmstadt, Germany), 2 mg·mL^−1^ hyaluronidase (H3506 Sigma‐a), and 2 mg·mL^−1^ BSA (A9418) until tissue was almost fully dissociated (30 min – 2 h at 37 °C), filtered through 100‐µm mesh filter (Falcon‐352360), and washed twice prior to cryopreservation in DMSO. The sentinel nodes were minced, filtered through 70‐µm filter (Falcon‐431751), washed twice, and cryopreserved in DMSO.

### 2.3 Mass cytometry

The cryopreserved single‐cell samples were thawed, and viability was measured by tryphan blue staining to ensure a minimum of 500 000 live cells per sample. In each run, a control sample of peripheral blood mononuclear cells (PBMC) spiked with equal amounts of the breast cancer cell lines BT474 (ATCC®HTB‐29™) and MB‐MBA 231 (ATCC®HTB‐26™) was stained and run in parallel with the patient samples (the control samples demonstrated overall high antibody staining consistency (Fig. S1)). Cells were stained with surface antibodies (Table S2) for 30 min, followed by wash and fixation (1.6% PFA) for 12 min prior to cell membrane permeabilization with 100% methanol (−20 °C). The cells were stored in methanol at −20 °C for at least 12 h and washed twice prior to intracellular staining for 25 min. The cells were then stained with Cell‐ID intercalator Ir (Fluidigm, San Fransisco, CA, USA) for 20 min and washed in PBS twice and then in water. The cells were resuspended in water and EQ‐beads and analyzed on a CyTOF 2 (Fluidigm).

### 2.4 Activation of T‐cell signaling and flow cytometry

T‐cell receptor (TCR) activation (a‐TCR): anti‐CD3 and anti‐CD28 biotin‐labeled antibodies were used at 5 μg·mL^−1^, and avidin (Thermo Fischer) was used at 50 μg·mL^−1^. Cells were stained using fluorochrome‐coupled antibodies (Table S3). Brilliant Stain Buffer (BD Biosciences, Franklin Lakes, NJ, USA) was used as staining buffer. Pacific Blue (Life Technologies, Carlsbad, CA, USA) was used for fluorescent barcoding of cells in phosphoflow cytometry analysis. In phenotype studies, cells were stained with Alexa Fluor 750 dye (Thermo Fisher Scientific Inc, Waltham, MA, USA) to exclude dead cells from analysis.

The activation of signaling and detection by phosphoflow were performed as described [23]. Specimens were thawed and cells were rested at 37 °C for 4 h, before redistribution into v‐bottomed 96‐well plates followed by 20 min of rest. Signaling was activated by a‐TCR for 1 or 4 min and stopped by adding 1.6% PFA, followed by permeabilization in ice‐cold methanol. After rehydration, cells were “barcoded” with Pacific Blue and stained with antibodies as previously described. The samples were collected on a LSR II flow cytometer (BD Biosciences). Relative phosphorylation changes (fold changes) were calculated using arcsinh transformation of median fluorescence intensity.

### 2.5 Immunohistochemistry and immunofluorescence

Formalin‐fixed paraffin‐embedded (FFPE) tissue from lymph nodes was sectioned (4 µm) and stained for hematoxylin and eosin (HE) and IHC‐stained for CD4 (4B12, Thermo Fisher), CD8 (NCL‐CD8‐4B11, Leica, Leica Biosystems, Wetzlar, Germany), and AE1/AE3 (cytokeratin CK1‐8, 10, 14‐16 and 19, M3515, DAKO, GmbH, Jena, Germany). Immunofluorescence was performed with high PH (Envision ™ FLEX target retrieval solution, DM828, DAKO) antigen retrieval and stained with CD8 ((C8/144B, cat.nr: 372902 BioLegend, San Diego, CA, USA)+ biotin (Gt anti‐mouse, M30115, Invitrogen, Thermo Fisher Scientific, MA, USA)+ streptavidin‐conjugated AF488 (S11223, Invitrogen)) and FoxP3 (236A/E7,ab20034 Abcam + goat‐anti‐Mouse AF594 (A21125, Invitrogen)) and counterstained with DAPI (Invitrogen™ ProLong™ Gold Antifade Mountant with DAPI, P36941, Fisher Scientific). The stainings were performed according to manufacture procedures. The sections were photographed in a Zeiss Axiovision Imager M1 microscope equipped with Axiocam MRc and Axiocam MRrm.

### 2.6 Data analysis

Mass cytometry data were normalized using the cytof^®^ Software 6.7 (Fluidigm) version 6.7.1014. Data analysis including expert gating, viSNE, and CITRUS was performed on the cloud‐based analysis platform Cytobank. Settings for CITRUS analysis: Event Sampling: Max per file, 5000 events; Minimum cluster size: 5%; correlative—significance analysis of microarrays (SAM); Statistics channels: Casp3, CD28, CD38, CD69, HLA‐DR, Ki‐67, pSTAT1, TIGIT. PD‐1 was not included in the CITRUS due to batch variation (high background) in one run. PD‐1 was, however, included in the inspection of marker expression in manually gated population, excluding the affected batch.

ImmunoPath (Room4 Group Ltd, Crowborough, UK) [24] software was used for objective scoring of IHC images. Extra‐ and intratumoral areas were drawn manually based on the expression of AE1/AE3. The field fraction function, calculating positive and negative pixels across the whole section, was used to calculate the positivity score for CD4 and CD8. We identified concordant results between mass cytometry and IHC quantification ranging from 51% to 99% (Table S4).

### 2.7 Statistics

The statistical language R [25] and GraphPad [26] were used for hierarchical clustering, boxplot, and statistical testing. Kruskal–Wallis nonparametrical test with Bonferroni correction, followed by Dunn’s *post hoc* test, was performed to calculate the statistical differences between sample groups. Spearman’s rank correlation was used for regression score. One‐tailed *t*‐test was used to calculate differences in tumor burden. Paired *t*‐test was used to calculate differences in expression between small sample groups.

## 3 Results

### 3.1 Identification of tumor cells in lymph nodes by mass cytometry

**Fig. 1:**
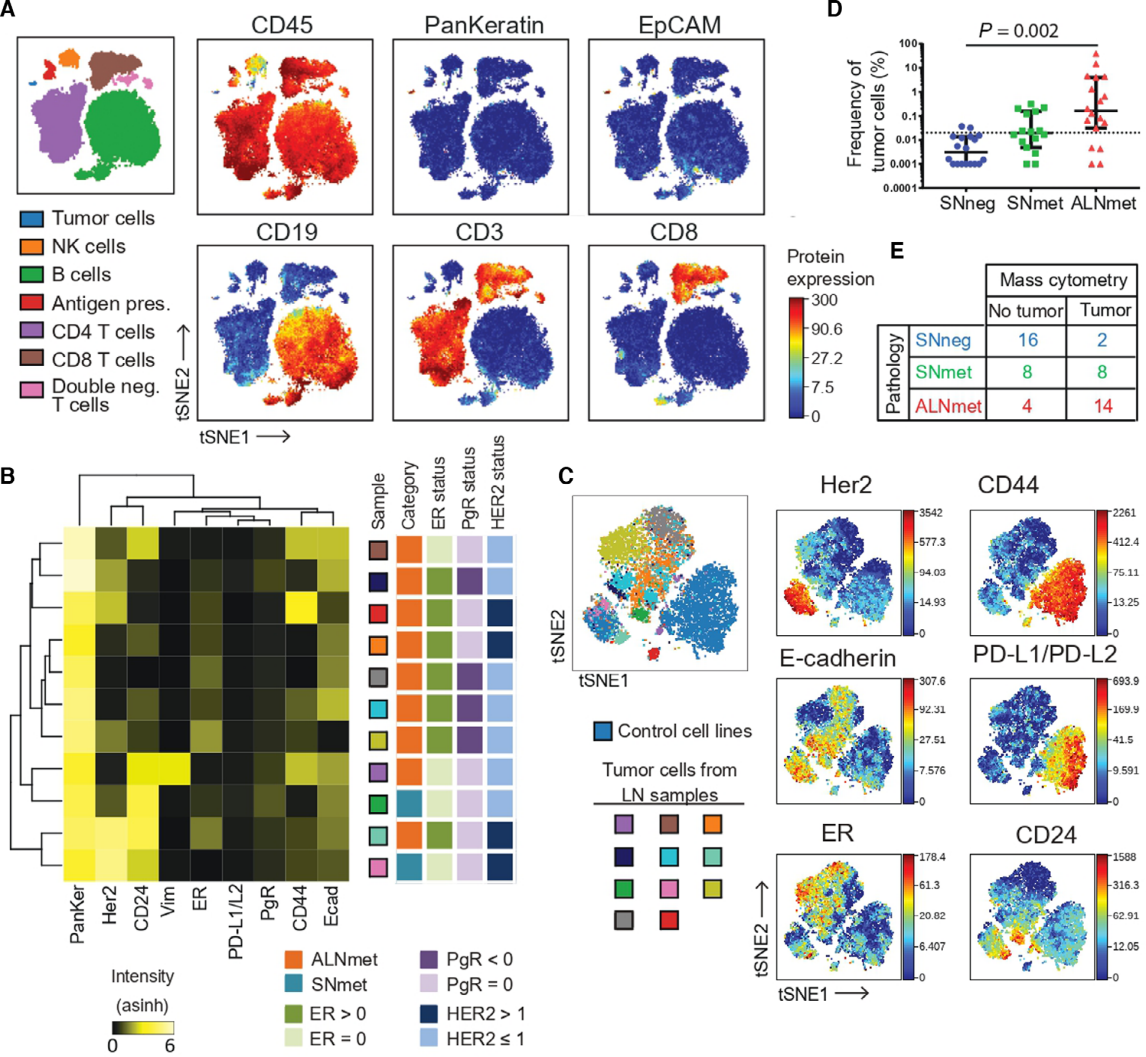
Simultaneous characterization of immune cells and tumor cells. Single‐cell suspensions of lymph nodes from 52 breast cancer patients were analyzed by mass cytometry. (A) viSNE map of all lineage markers from one representative SNmet (metastatic sentinel node) sample. Each dot in the viSNE map represents a single cell, and the position of each cell is based on similarity of the markers included in the viSNE analysis. The color of the dot represents expression intensity of markers as indicated. More markers are shown in Fig. S2. (B, C) The tumor cells from all samples with more than 100 live tumor cells (*n* = 11) were analyzed by hierarchical clustering (B) and viSNE (C). (D) Tumor cell percentage for each sample compared with pathology classification. The dotted line marks the selected threshold to call a sample as negative; 0.02%. Samples with no tumor cells are set to 0.001%. Lines represent median with ± 95% CI. *P*‐values from Kruskal–Wallis test and Dunn’s post hoc test. (E) Cross‐table showing concordance between pathology scoring and mass cytometry detection of tumor cells.

Our material consisted of lymph nodes from 52 patients with primary operable non‐metastatic breast cancer (Table S1). For simultaneous profiling of tumor and immune cells, we established a 37‐parameter antibody panel with markers for identification and subtyping of tumor cells, immune cell phenotypes, and functional assessment (Fig. S2A). The panel identified both tumor cells and a diversity of immune cell populations as expected (Fig. 1A, Figs S2 and S3). Eleven cases had sufficient number of metastatic tumor cells for further analysis, and the expression patterns of ER, PgR, and HER2 in the metastatic tumor cells reflected the pathology assessment of the corresponding primary tumors (Fig. 1B). Hierarchical clustering and viSNE analysis of mass cytometry data revealed intertumoral heterogeneity in the expression of stem cell markers CD44 and CD24 across the 11 samples, whereas PD‐L1/PD‐L2 expression was low/undetectable in all cases (Fig. 1B). The viSNE analysis further showed limited intratumoral variation (Fig. 1C). These results could be biased due to low numbers of tumor cells per sample analyzed or might reflect that only specific subpopulations of the primary tumor are capable of metastasizing, resulting in lower heterogeneity of metastasizing tumor cells [27].

By standard pathology examination, the lymph nodes were categorized as metastatic axillary lymph nodes (ALNmet, *n* = 18), metastatic sentinel nodes (SNmet, *n* = 16), or non‐metastatic sentinel nodes (SNneg, *n* = 18). These samples were chosen to represent all molecular subtypes and a range of tumor burden, and we observed undetectable/very low tumor cell percentage in SNneg, higher in some of the SNmet samples and significantly highest in ALNmet (Fig. 1D). This was expected as sentinel nodes are sampled only when there is no suspected axillary metastasis, and pathology revision validated significantly lower tumor burden in SNmet samples compared with ALNmet samples (*P* = 0.04 by *t*‐test; Table S1). By selecting a threshold of 0.02% tumor cells per total nucleated cells to classify samples as tumor positive, we observed 73% (38/52) concordance between mass cytometry and pathology scoring (Fig. 1E). The largest discrepancy in classification was seen for SNmet samples, where the mass cytometry analysis only detected tumor cells in 8 out of 16 samples. Interestingly, mass cytometry analysis identified tumor cells in 2 out of 18 SNneg samples (Fig. 1D,E). The discordance between pathology classification and mass cytometry might be due to variable distribution of tumor cells in the lymph node halves, one used for pathology review and the other used for mass cytometry. In addition, tumor cells are fragile and can more easily be destroyed during processing into single‐cell suspension, leading to an underrepresentation of tumor cells. Two prior studies re‐evaluated lymph nodes scored as tumor negative by pathology and found tumor cells in 20% and 28% of lymph nodes by RT‐PCR and by ten‐level IHC staining, respectively [18, 28]. Together, the 52 samples represent a continuous spectrum of tumor burden, reflecting different stages of the metastatic process. This facilitates investigation of immune response dynamics from small deposits to manifest metastases in lymph nodes.

### 3.2 Skewing of T‐cell subset distribution toward memory CD8 T Cells in Metastatic Lymph Node Samples

**Fig. 2:**
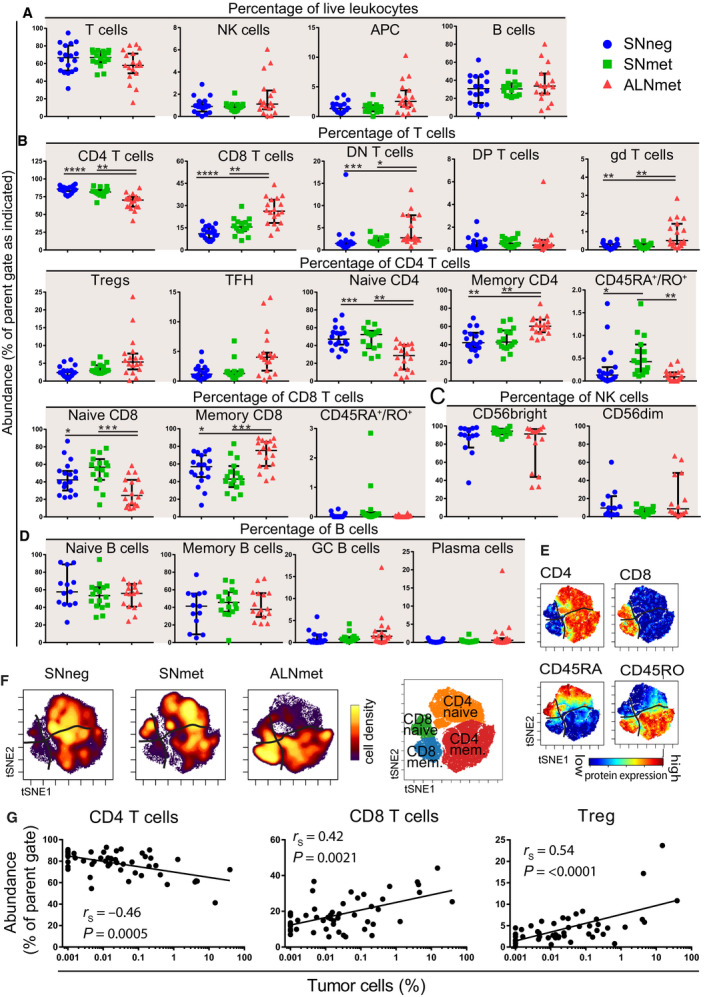
The T‐cell composition in ALNmet samples is skewed toward CD8 T cells and a memory phenotype. Frequency of immune subsets in lymph nodes analyzed by mass cytometry. Lines represent median with ± 95% CI. * indicates significance with Kruskal–Wallis test (Bonferroni corrected, 23 tests) and Dunn’s post hoc test. *n* = 52 unless otherwise stated. *P < 0.05, **P < 0.01 ***P < 0.001, ****P < 0.0001. (A) Main immune subsets calculated as percentage of total live CD45^+^ cells. APC = antigen‐presenting cells. (B) T‐cell subsets, calculated as percentage of total T cells, CD4 T cells, CD8 T cells, or DN T cells as indicated. DN = double negative, DP = double positive. (C) NK (natural killer)‐cell subsets, calculated as percentage of total NK cells. Samples with < 100 NK cells were excluded. *n* = 41. (D) B‐cell subsets, calculated as percentage of total B cells. Some samples were excluded due to suboptimal staining. *n* = 45 naive/memory. *n* = 49 GC (germinal center/plasma cells. (E, F) viSNE map of T cells concatenated from all lymph node samples based on the expression of CD4, CD8, CD45RA, CD45RO, TCRγδ, FoxP3, CD25, PD‐1. (F) Density maps show SNneg (non‐metastatic sentinel node), SNmet (metastatic sentinel node), and ALNmet (axillary metastatic lymph node) samples concatenated separately. Color indicated intensity of marker expression. (G) Correlation analysis of abundance of immune subsets vs tumor percentage (Spearman`s rank correlation). The straight line was found by linear regression of abundance of immune cells on the logarithm of tumor abundance. *r*_S_ = Spearman correlation coefficient.

To address potential impact of metastatic tumor cells on immune cell distribution, we gated CD45‐positive cells into 23 different immune cell populations (Fig. S3) and compared the cell type distribution between SNneg, SNmet, and ALNmet samples (Fig. 2A–D). We found statistically significant differences in the frequencies of nine T‐cell subsets, whereas no differences were found within B‐cell and NK‐cell subsets. Almost all significant differences were observed between SNneg/met and ALNmet (Fig. 2A–D, Table S5). This included skewed CD4/CD8 T‐cell ratio with increased frequencies of CD8 T cells in ALNmet compared with SNneg and SNmet samples. Furthermore, whereas SNneg and SNmet samples had relatively equal fractions of naïve and memory CD4 and CD8 T cells, this balance was highly skewed toward memory T cells in ALNmet samples (Fig. 2B). The shift in distribution from naïve to memory T cells in ALNmet samples was further visualized by viSNE analysis (Fig. 2E), and the corresponding cell density plots demonstrate that SNneg and SNmet samples primarily contained CD4 T cells with similar fraction of naïve and memory T cells, whereas ALNmet samples were dominated by memory CD8 and CD4 T cells (Fig. 2F). Some additional cell populations including APC, Tregs, TFH, and plasma cells were more frequent in ALNmet compared with SNneg and SNmet samples, although this did not reach statistical significance when correcting for multiple testing (Table S5).

The only significant difference between SNneg and SNmet was a small population of CD45RA^+^CD45RO^+^ CD4 cells that was slightly increased in SNmet compared with SNneg and ALNmet samples (Fig. 2B). Limited data exist on the role of CD45RA^+^CD45RO^+^ CD4 T cells, which are the cellular state when unprimed CD4 T cells become active and can express both markers simultaneously for a short period. The small differences between SNneg and SNmet samples could indicate that a certain tumor burden must be reached before changes in immune cell distribution can be detected. However, by plotting tumor cell frequencies against the fraction of immune cell subsets, we found a significant positive association between tumor burden and increased frequencies of CD8 T cells and Tregs, whereas the frequency of CD4 T cells was inversely associated with tumor burden (Fig. 2G). Collectively, these data demonstrated a strong shift in immune cell distribution in samples with higher tumor burden.

### 3.3 Phenotypic changes associated with immunosuppression in ALNmet samples

**Fig. 3:**
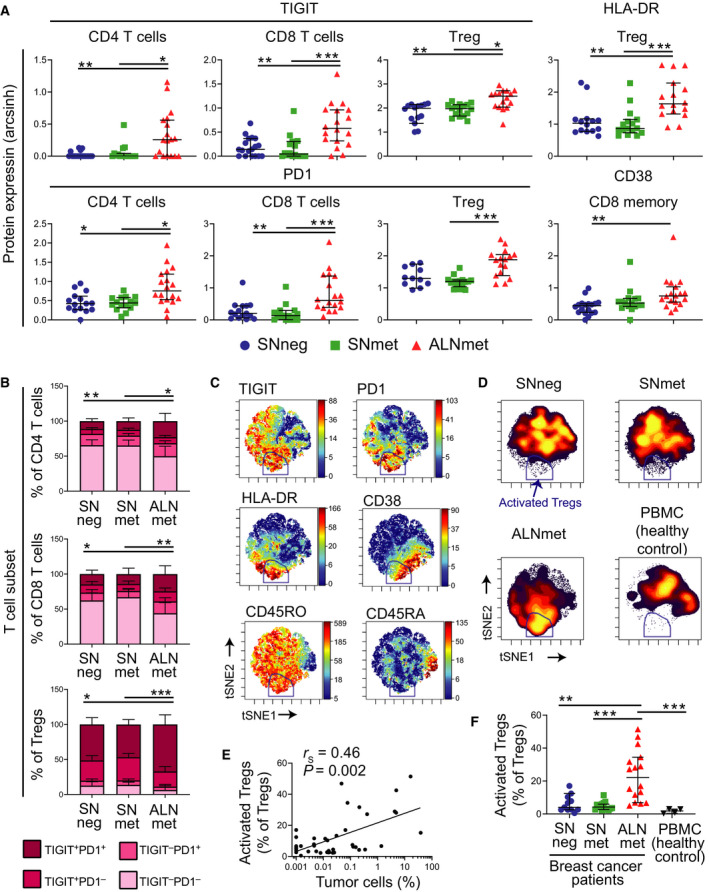
ALNmet samples have increased presence of TIGIT+PD‐1 + T cells and phenotypically activated Tregs. (A) A CITRUS analysis identified differential expression of exhaustion markers and Treg activation markers in T cells from ALNmet samples compared with SNneg (non‐metastatic sentinel node) and SNmet (metastatic sentinel node) samples (see also Fig. S4A–C and Table S6). The median intensity (arcsinh‐transformed) of such markers is shown for manually gated populations. Lines represent median with 95% CI. *P < 0.05, **P < 0.01 ***P < 0.001 in Kruskal–Wallis test (Bonferroni corrected, 13 tests) and Dunn’s post hoc test. (B) Percentage positive cells falling into a quadrant gate set on PD‐1 and TIGIT, bars represent mean ± SD. Statistical testing performed only on percentage of double‐positive cells. *P < 0.05, **P < 0.01, ***P < 0.0001 in Kruskal–Wallis test and Dunn`s *post hoc* test. (C‐F) A viSNE analysis was performed with activation markers (TIGIT, PD‐1, HLA‐DR, CD38, CD45RA, CD45RO) on Tregs from all samples with more than 100 Tregs, including PBMC control samples. A gate was set on the cells positive for all the activation markers, termed activated Tregs. Marker intensity for all events concatenated (C), and density maps of each sample type concatenated (D), percentage of activated Tregs against percentage of tumor cells and linear regression by Spearman (E). Percentage of activated Tregs in SNneg (non‐metastatic sentinel node), SNmet (metastatic sentinel node), and ALNmet (metastatic axillary lymph nodes) from breast cancer patients and PBMC from healthy controls. Lines represent median 96% CL. **P < 0.01, ***P < 0.0001 in Kruskal–Wallis test and Dunn`s post hoc test (F).

To investigate potential tumor cell‐induced functional alterations in T cells, we performed CITRUS [29] analysis using expression of eight markers including checkpoint receptor TIGIT, activation and differentiation markers (phospho‐STAT1, HLA‐DR, CD38, CD69, Ki‐67, CD28), and marker of apoptosis (cleaved‐caspase 3). This revealed seven clusters differing in the expression of at least one of the markers TIGIT, HLA‐DR, CD38, or CD69 (Fig. S4A,B). Five unique clusters representing different T‐cell subsets were annotated based on the expression pattern of lineage markers (Fig. S4C). Further inspection of the corresponding manually gated populations demonstrated that CD4 and CD8 T cells in ALNmet samples had increased expression levels of the exhaustion markers TIGIT and PD‐1, compared to SNneg and SNmet samples (Fig. 3A,B, Table S6). Memory CD8 T cells in ALNmet had elevated levels of the activation marker CD38, compared to SNneg samples. Of note, Tregs from ALNmet samples had significantly increased expression of TIGIT, HLA‐DR, and PD‐1 (Fig. 3A,B, Table S6). To study co‐expression of these markers, we performed the following: viSNE analysis on Tregs. This approach revealed a subset of mainly present in ALNmet samples consisting of cells co‐expressing PD‐1, TIGIT, HLA‐DR, CD38, and CD45RO (Fig. 3C–F), suggesting that these are activated Tregs [30, 31]. The frequency of activated Tregs was directly correlated with tumor burden (Fig. 3E). Collectively, the observed changes in ALNmet T cells suggest T‐cell recognition of tumor cells, but suppressed immunity through increased expression of TIGIT and PD‐1 in effector T cells and increased frequency of activated Tregs.

### 3.4 TIGIT‐expressing T cells display dysfunctional TCR signaling

**Fig. 4:**
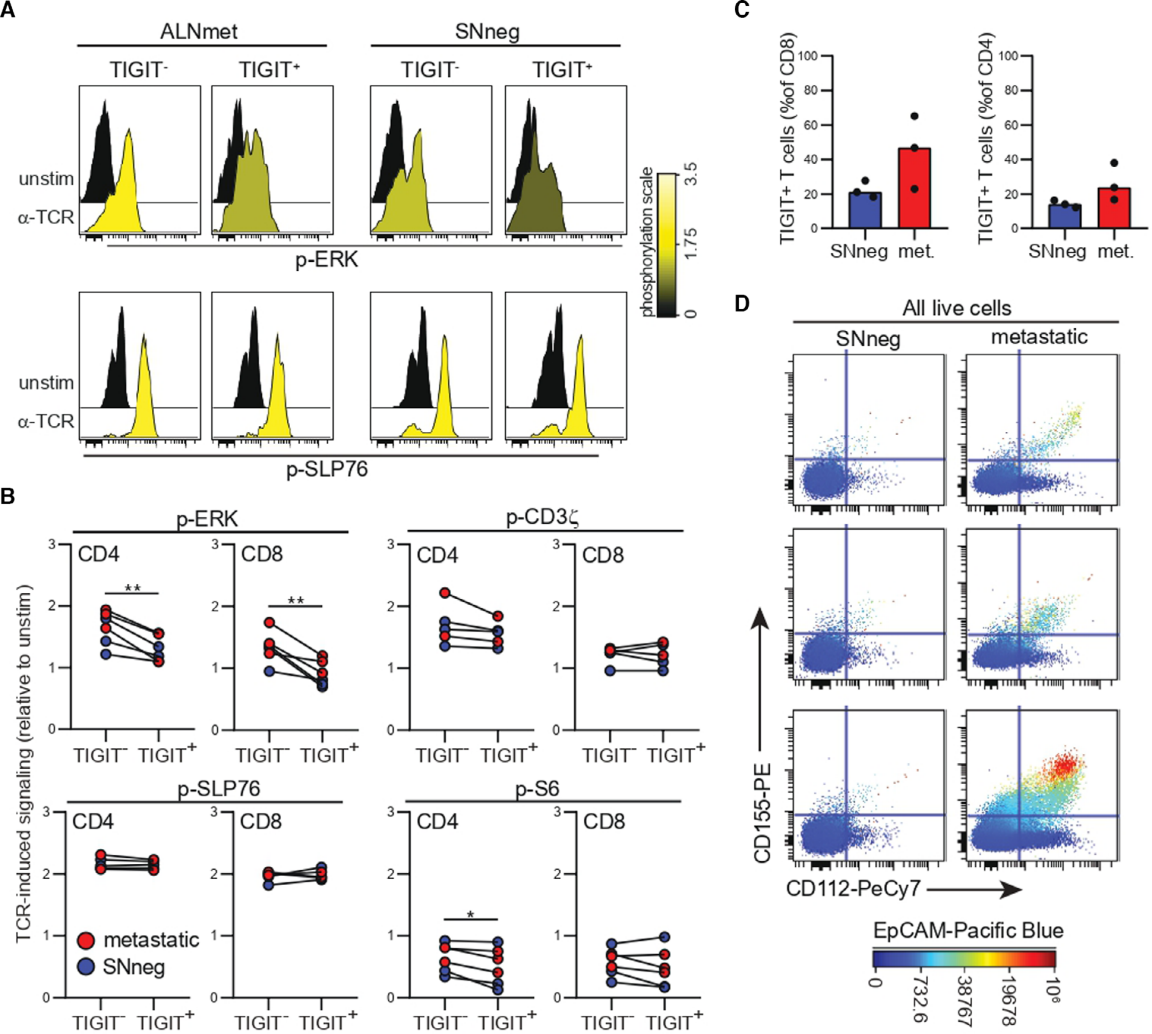
TIGIT+ T cells are dysfunctional. (A, B) TCR signaling was activated using anti‐CD3 and anti‐CD28 biotinylated antibodies, followed by avidin crosslinking in 3 SNneg (non‐metastatic sentinel node) and 3 metastatic lymph nodes (1 SNmet (metastatic sentinel node), 2 ALNmet (metastatic axillary lymph node)). Signaling was measured by phosphoflow cytometry and showed reduced p‐ERK signaling in TIGIT+ cells compared with TIGIT‐ cells. (A) Signaling in CD8+ T cells from two out of six representative samples. (B) TCR‐induced signaling in CD4 and CD8 T cells relative to unstimulated cells, shown as arcsinh median, *n* = 3 metastatic lymph nodes and *n* = 3 non‐metastatic lymph nodes. *P < 0.05, **P < 0.01 in paired t‐test. (C) Flow cytometry analysis of the same six samples confirmed higher TIGIT expression in T cells from metastatic samples by paired t‐test, nonsignificant. Bars represent median values. (D) The samples were also stained for TIGIT ligands CD155 and CD112. Cytograms display all live singlets of the six samples. Tumor cells and macrophages/monocytes (identified by CD4+CD3‐; Fig. S5A) expressed CD155 and CD112 in both non‐metastatic and metastatic lymph nodes, but increased presence of ligand+ cells in metastatic samples.

To address whether TIGIT expression in effector T cells was associated with dysfunction, we investigated the relationship between TIGIT expression and T‐cell receptor (TCR) signaling capacities of T cells from three metastatic lymph nodes and three SNneg samples using phosphoflow cytometry. This functional analysis revealed that TIGIT^+^ T cells were distinguished by lower TCR‐induced phosphorylation (p) of ERK, compared with TIGIT^‐^ cells (Fig. 4A,B). Strikingly, TCR‐induced p‐ERK was significantly reduced in TIGIT^+^ CD4 and CD8 T cells from both SNneg and metastatic lymph node samples, whereas pS6 was reduced in TIGIT^+^ CD4 T cells (Fig. 4B). In contrast, TCR proximal signaling, as determined by p‐SLP76 and p‐CD3ζ, was strong and comparable in TIGIT^+^ and TIGIT^‐^ T cells (Fig. 4B), indicating that TIGIT plays a role in dampening signaling in the distal part of the TCR signaling pathway.

Phenotypic analysis of the same samples confirmed a trend toward increased frequency of TIGIT‐expressing CD4 and CD8 T cells in ALNmet compared with SNneg (Fig. 4C). Tumor cells and macrophages expressed TIGIT ligands CD155 and CD112 (Fig. 4D, Fig. S5A). As TIGIT competes for ligand binding with the co‐stimulatory receptor CD226, co‐staining of the two receptors to identify their relationship was performed (Fig. S5B). There was a trend of increased frequency of TIGIT^+^CD226^‐^ cells and reduced frequency of TIGIT^‐^CD226^+^ cells among memory CD8 T cells in ALNmet compared with SNneg samples (Fig. S5C), indicating an imbalance between T‐cell co‐stimulation and co‐inhibition, tipping the balance further toward T‐cell dysfunction in metastatic lymph nodes. We also saw a correlation between tumor burden and TIGIT expression in CD4 and CD8 T cells (Fig. S5D,E). Collectively, these results demonstrated that TIGIT^+^ T cells have dysfunctional TCR signaling also in SNneg samples without detectable tumor cells. However, the higher frequency of TIGIT^+^ T cells in ALNmet samples together with the finding that metastatic tumor cells expressed TIGIT ligands suggests that tumor cells promote T‐cell dysfunction.

### 3.5 Spatial analysis of immune markers in metastatic lymph nodes

**Fig. 5:**
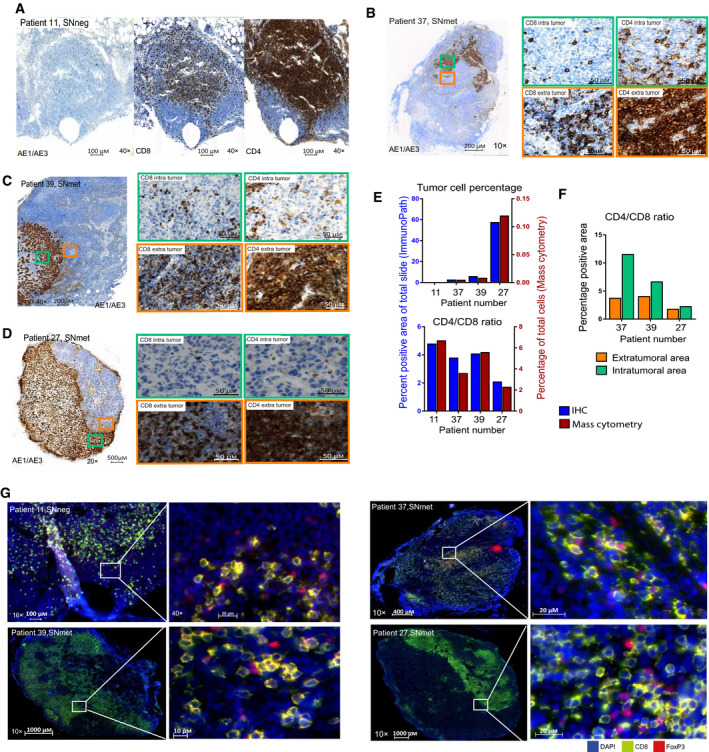
The topological distribution of CD4‐ and CD8‐positive cells in lymph nodes with no, small, intermediate, and large metastatic tumor burden. (A–D) One SNneg (non‐metastatic sentinel node) (patient 11) and three SNmets (metastatic sentinel nodes) (patients 37,39, and 27) were IHC stained with AE1/AE3, CD4, and CD8. Magnified area of extratumoral (orange) and intratumoral (green) regions stained with CD8 and CD4 of lymph nodes with small (B), intermediate (C), and large (D) tumor burden. Scales and magnifications are shown in images. (E, F) A scoring system, ImmunoPath (with field fraction function measuring positive and negative pixels was used to calculate the percentage positive areas) was used to objectively score IHC‐positive areas. (E) Histograms comparing tumor cell percentage and CD4/CD8 ratio measured by IHC (left axis) and mass cytometry (right axis) performed on cells from the same lymph nodes. (F) Comparison of CD4/CD8 ratio in intratumoral and extratumoral areas of the metastatic lymph node analyzed separately by ImmunoPath. (G) Immunofluorescence staining with CD8 (green) and Treg (FoxP3, red) and counterstained with DAPI on non‐metastatic sentinel node and metastatic sentinel nodes (same samples as A‐D) Scales and magnifications are shown in images.

To investigate whether the observed changes in immune cell composition applied only to immune cells in direct contact with tumor cells, we analyzed formalin‐fixed paraffin‐embedded tissue (FFPE) from one SNneg and three SNmet samples by IHC where the latter represented lymph nodes with different sizes of tumor burden. Sections were stained for hematoxylin and eosin (HE), AE1/AE3 (cytokeratins 1‐8, 10, 14‐16, and 19 used as tumor cell marker), CD4, and CD8 as illustrated in Fig. 5A–D. We observed AE1/AE3 staining in all three SNmet samples and identified scattered CD4 and CD8 staining within the tumor area (Fig. 5B–D). For an objective assessment of the number of CD4 and CD8 T cells, the IHC‐stained FFPE slide images were automatically scored by the ImmunoPath software. IHC/ImmunoPath and mass cytometry revealed similar trends for tumor cell percentage and CD4/CD8 ratio (Fig. 5E). In the three metastatic lymph nodes, CD4 and CD8 staining were assessed separately in intratumoral and extratumoral areas and a CD4/CD8 ratio above one was found in both areas (Fig. 5F). Interestingly, the ratio was lowest in the patient sample with the highest tumor burden (patient 27) due to skewing toward CD8 cells. The skewing was even more pronounced in the intratumoral than the extratumoral area, suggesting that the shift in immune cell distribution is most prominent in areas where immune cells are in direct contact with tumor cells.

We next studied the location of Tregs in relation to CD8 T cells by dual immunofluorescence from the same samples (Fig. 5G). Tregs were observed in the vicinity of CD8 T cells in extratumoral areas in all four lymph nodes. Together, our data show that metastatic tumor cells attract more CD8 T cells, and imply suppression of CD8 T cells by the presence of Tregs.

## 4 Discussion

Lymph nodes serve two critical functions; they filter lymph fluid and have a key role in pathogen defense by orchestration of adaptive immunity. The sentinel node is the first lymph node draining the lymphatic from the breast and can thus be expected to be the site of an early immune response toward tumor cells. Recent advancement in immunotherapy has revealed new opportunities in treating cancer, yet immunotherapy is not part of an adjuvant treatment regimen. However, early intervention with immune checkpoint blockade might be relevant if immunosuppression is prominent already at the early steps of the metastatic process. By analyzing lymph nodes representing the full spectrum of the metastatic process, from uninvolved nodes to micro‐metastatic deposits and fulminant metastases, we identified dynamic differences in T‐cell subset composition and their functional status, reflecting the size of the metastases.

Key regulators of the metastatic process in breast cancer are still largely unknown, but one hypothesis is that tumors initiate a pre‐metastatic niche in metastatic sites prior to metastasizing. A comparison of sentinel nodes and distant lymph nodes in early cervical cancer identified a significant increase in CD8 T cells, and expression of FoxP3 and PD‐1 in SN compared with distant lymph nodes [32]. An altered profile of lymphatic endothelial cells was observed in tumor‐draining lymph nodes compared with naïve lymph nodes in models of breast cancer and melanoma [33]. These findings indicate that sentinel nodes are affected early in the metastatic process and might be primed as metastatic sites even before tumor cells arrive. This could be facilitated by intratumoral dendritic cells, engulfing fragments of tumor cells and then travel to the sentinel node for antigen presentation to T cells. In our analysis of single‐cell suspensions, we observed a correlation between changes in T‐cell immune profiles and tumor burden. Of note, if these changes primarily are caused by direct interaction between tumor cells and T cells via TCR recognition of neoantigens presented by tumor cells MHC, the changes in lymph nodes with small tumor burden will be less obvious in bulk analysis. Supporting this view, we observed that the skewing in CD4/CD8 ratio was more prominent within the tumor area than in the extratumoral area. Changes in T cells upon tumor cell interaction in lymph nodes with even small metastatic deposits might therefore be visible by analyzing tissue arrays of lymph nodes. Single‐cell profiling of non‐metastatic and metastatic lymph nodes by multiplex spatial methods such as imaging mass cytometry could provide further insight into the metastatic process, by obtaining high‐dimensional maps of the early tumor cell immune cell interactions. The method has been successfully used to characterize the heterogeneity in cellular composition across different tumor tissues, including variability in immune cell infiltration in primary breast cancer tumors [16, 34]. Primary tumors characterized by communities with high T‐cell infiltration or high macrophage infiltration had better patient outcomes [34], although these findings warrant validation in larger cohorts of breast cancer subtypes. Based on this, we believe that high‐dimensional spatial analysis of metastatic lymph nodes could provide further insight into the metastatic process, by obtaining high‐dimensional maps of the early tumor cell immune cell interactions.

Immune suppression is a critical stage for metastatic tumor cells to escape immune surveillance. We discovered that the presence of metastatic tumor cells was associated with higher fraction of T cells expressing the exhaustion markers PD‐1 and TIGIT. By combined detection of TIGIT, T‐cell markers and the phosphorylation status of kinases and adaptor proteins post‐TCR activation, we found a clear correlation between TIGIT expression and defect in TCR‐induced p‐ERK in CD4 and CD8 T cells and in TCR‐induced pS6 in CD4 T cells. Reduced pS6 suggests attenuation of mTOR pathway and is indicative of CD4 T‐cell exhaustion [35]. Our finding that TIGIT expression is linked to T‐cell exhaustion is consistent with previous studies, demonstrating that TIGIT marks dysfunctional T cells with reduced TCR signaling, attenuated TCR‐driven activation signals, and reduced production of inflammatory cytokines [23, 36, 37]. Changes in T cells in metastatic lymph nodes included downregulation of the co‐stimulatory receptor CD226, which competes with TIGIT for ligand binding, favoring co‐inhibition over co‐stimulation. Collectively, this demonstrates higher degree of T‐cell exhaustion in metastatic lymph nodes. Further, Tregs are key players in promoting progression and maintaining a tumor friendly environment by suppressing activation and expansion of effector T cells. The tumor microenvironment produces factors that facilitate increased recruitment of Tregs and/or conversion of conventional T cells into Tregs. An increased number of Tregs are found in several tumor types, including breast and ovarian cancer [38, 39]. There is increasing evidence for molecular and functional heterogeneity in the circulating as well as tissue‐resident pool of Tregs [31, 40]. We found that higher frequency of Treg and higher co‐expression of HLA‐DR, PD‐1, CD38, TIGIT, and CD45RO were correlated with tumor burden. Several lines of evidence support that Tregs expressing checkpoint receptors have higher suppressive activity. TIGIT+ Tregs had higher suppressive capacity than TIGIT‐ Tregs and a preference to inhibit Th1 and Th17 CD4 T cells [30]. Intratumoral Tregs from non‐small‐cell lung cancer and colorectal cancer had higher expression of several checkpoint receptors including CTLA‐4, TIGIT, and ICOS and had stronger capacity to suppress proliferation of autologous effector T cells as compared to Tregs purified from peripheral blood or normal tissue [41]. The presence of these highly suppressive Tregs could be a key mechanism for suppressing effective anti‐tumor immunity. Our findings are in line with a recent publication showing that tumor‐draining lymph nodes with metastatic breast cancer cells had more effector Tregs and less naïve Tregs compared with tumor‐negative lymph nodes [42]. Investigation of Treg subsets in blood and primary tumor biopsies from breast cancer patients demonstrated phenotypic similarity between Treg fraction II in blood, corresponding to activated Tregs, and intratumoral Tregs with high expression of CTLA‐4, TIGIT, and ICOS [43].

In metastatic lymph nodes, we observed Tregs to be in direct contact with CD8 T cells within extratumoral areas. Collectively, our data support the hypothesis that tumor cells escape immunity by direct or indirectly promoting T‐cell exhaustion and by recruiting more Tregs to the lymph node.

## 5 Conclusion

This is to our knowledge the first study investigating the frequency of more than 20 immune cell types simultaneously across a continuous spectrum of tumor progression in lymph nodes from breast cancer patients. Our study indicates immune suppression to occur already at early stages of local spread of breast cancer and changes in immune cell frequencies reflected tumor burden. Our observation of increased frequency of Tregs with an activated phenotype combined with dysfunctional TIGIT‐expressing CD4 and CD8 T cells suggests dampening of anti‐tumor T‐cell responses in lymph nodes with established metastases. TIGIT is an emerging checkpoint, and TIGIT targeting is likely to be most efficient when combined with other immunotherapies [44]. Preclinical *in vivo* studies using colon and breast cancer models demonstrated strong synergistic anti‐tumor effects when anti‐TIGIT blockade was combined with targeting of PD‐1 [45]. Six anti‐human TIGIT antibodies have been developed for clinical use, and more than fifteen clinical trials testing anti‐TIGIT therapy alone or in combinations are ongoing or will soon start recruitment in advanced/metastatic solid cancers. The combination of TIGIT and PD‐L1 blockade was recently reported to have impressive effect in non‐small‐cell lung cancer [46] and was recently approved as breakthrough therapy designation by FDA. Our study indicates that TIGIT and PD‐1 are relevant targets for checkpoint inhibition in breast cancer that warrants testing also in the non‐metastatic setting to prevent metastasis. On the flip side, as hyperproliferative disease has been observed in some patients in response to checkpoint blockade with PD‐1 and attributed to the presence of intratumoral PD‐1+ Tregs [47] the therapeutic effect on intratumoral Tregs needs further investigation.

## Conflict of interest

The authors declare no conflict of interest.

## Author contributions

IHR conceptualized, curated the data, involved in formal analysis, investigated, contributed to project administration, visualized, and wrote—review and editing. KH conceptualized, curated the data, involved in formal analysis and funding acquisition, investigated, contributed to project administration, visualized, and wrote—review and editing. SEJ curated the data, involved in formal analysis, investigated, visualized, and wrote—review & editing. WK investigated, provided software, and wrote—review & editing. HED provided software, and wrote—review & editing. OCL wrote—review & editing. ES and ØG provided resources and wrote—review & editing. MLR curated the data and wrote—review & editing. OSBREAC provided resources. JHM conceptualized, involved in funding acquisition and project administration, investigated, supervised, and wrote—review & editing. HGR conceptualized, curated the data, involved in funding acquisition and project administration, investigated, supervised, and wrote—review & editing.

### Peer Review

The peer review history for this article is available at https://publons.com/publon/10.1002/1878‐0261.13047.

## Supporting information


**Fig. S1**. Consistency of staining.
**Fig. S2**. Mass cytometry antibody panel.
**Fig. S3**. Gating scheme.
**Fig. S4**. Citrus analysis on T cells.
**Fig. S5**. Expression of TIGIT and TIGIT related proteins.
**Table S1**. Clinical and histopathological parameters.
**Table S2**. Mass cytometry antibody panel.
**Table S3**. Antibodies used for Flow Cytometry analysis and TCR activation.
**Table S4**. Comparison of CD4 and CD8 quantification in four patient samples (Fig. 5).
**Table S5**. p‐values for abundance analysis (Fig. 2).
**Table S6**. p‐values for marker expression analysis (Fig. 3).

## Data Availability

Mass cytometry data supporting the findings of this study are openly available https://github.com/ingahrye/SNproject.git

## References

[1] Bray F , Ferlay J , Soerjomataram I , Siegel RL , Torre LA & Jemal A (2018) Global cancer statistics 2018: GLOBOCAN estimates of incidence and mortality worldwide for 36 cancers in 185 countries. CA Cancer J Clin 68, 394–424. 30207593 10.3322/caac.21492

[2] Hu Z , Fan C , Oh DS , Marron JS , He X , Qaqish BF *et al*. (2006) The molecular portraits of breast tumors are conserved across microarray platforms. BMC Genom 7, 96. 10.1186/1471-2164-7-96 PMC1468408 16643655

[3] Curtis C , Shah SP , Chin SF , Turashvili G , Rueda OM , Dunning MJ , Speed D , Lynch AG , Samarajiwa S , Yuan Y *et al*. (2012) The genomic and transcriptomic architecture of 2,000 breast tumours reveals novel subgroups. Nature 486, 346–352. 22522925 10.1038/nature10983 PMC3440846

[4] Loi S , Michiels S , Salgado R , Sirtaine N , Jose V , Fumagalli D , Kellokumpu‐Lehtinen PL , Bono P , Kataja V , Desmedt C *et al*. (2014) Tumor infiltrating lymphocytes are prognostic in triple negative breast cancer and predictive for trastuzumab benefit in early breast cancer: results from the FinHER trial. Ann Oncol 25, 1544–1550. 24608200 10.1093/annonc/mdu112

[5] Luen S , Virassamy B , Savas P , Salgado R & Loi S (2016) The genomic landscape of breast cancer and its interaction with host immunity. Breast 29, 241–250. 27481651 10.1016/j.breast.2016.07.015

[6] Sidhu B , Bowers L , Noonan G , Murison P , Liu G , Ly K , Turner J , Vriends K & St‐Pierre C (2018) American Joint Committee on Cancer (AJCC) cancer staging eighth edition: prognostic vs anatomic path stage group for breast cancer. J Registry Manag 45, 78–79. 31533133

[7] Kim T , Giuliano AE & Lyman GH (2006) Lymphatic mapping and sentinel lymph node biopsy in early‐stage breast carcinoma. Cancer 106, 4–16. 16329134 10.1002/cncr.21568

[8] Veronesi U , Paganelli G , Viale G , Luini A , Zurrida S , Galimberti V , Intra M , Veronesi P , Robertson C , Maisonneuve P *et al*. (2003) A randomized comparison of sentinel‐node biopsy with routine axillary dissection in breast cancer. N Engl J Med 349, 546–553. 12904519 10.1056/NEJMoa012782

[9] Giuliano A (2017) Effect of axillary dissection vs no axillary dissection on 10‐year overall survival among women with invasive breast cancer and sentinel node metastasis: the ACOSOG Z0011 (Alliance) randomized clinical trial. JAMA 318, 918. 28898379 10.1001/jama.2017.11470 PMC5672806

[10] Weaver DL , Ashikaga T , Krag DN , Skelly JM , Anderson SJ , Harlow SP , Julian TB , Mamounas EP & Wolmark N (2011) Effect of occult metastases on survival in node‐negative breast cancer. N Engl J Med 364, 412–421. 21247310 10.1056/NEJMoa1008108 PMC3044504

[11] Andersson Y , Frisell J , Sylvan M , De Boniface J & Bergkvist L (2010) Breast cancer survival in relation to the metastatic tumor burden in axillary lymph nodes. J Clin Oncol 28, 2868–2873. 20458033 10.1200/JCO.2009.24.5001

[12] Groom JR (2019) Regulators of T‐cell fate: integration of cell migration, differentiation and function. Immunolo Rev 289, 101–114. 10.1111/imr.12742 30977199

[13] Yarchoan M , Johnson BA III , Lutz ER , Laheru DA & Jaffee EM (2017) Targeting neoantigens to augment. Nat Rev Cancer 17, 209–222. 28233802 10.1038/nrc.2016.154 PMC5575801

[14] Chung W , Eum HH , Lee H‐O , Lee K‐M , Lee H‐B , Kim K‐T , Ryu HS , Kim S , Lee JE , Park YH *et al*. (2017) Single‐cell RNA‐seq enables comprehensive tumour and immune cell profiling in primary breast cancer. Nat Commun 8, 15081. 28474673 10.1038/ncomms15081 PMC5424158

[15] Azizi E , Carr AJ , Plitas G , Cornish AE , Konopacki C , Prabhakaran S , Nainys J , Wu K , Kiseliovas V , Setty M *et al*. (2018) Single‐cell map of diverse immune phenotypes in the breast tumor microenvironment. Cell 174, 1293–1308.e36. 29961579 10.1016/j.cell.2018.05.060 PMC6348010

[16] Wagner J , Rapsomaniki MA , Chevrier S , Anzeneder T , Langwieder C , Dykgers A , Rees M , Ramaswamy A , Muenst S , Soysal SD *et al*. (2019) A single‐cell atlas of the tumor and immune ecosystem of human breast cancer. Cell 177, 1330–1345. 30982598 10.1016/j.cell.2019.03.005 PMC6526772

[17] Keren L , Bosse M , Marquez D , Angoshtari R , Jain S , Varma S , Yang S‐R , Kurian A , Van Valen D , West R *et al*. (2018) A structured tumor‐immune microenvironment in triple negative breast cancer revealed by multiplexed ion beam imaging. Cell 174, 1373–1387.e19. 30193111 10.1016/j.cell.2018.08.039 PMC6132072

[18] Matsuura K , Yamaguchi Y , Ueno H , Osaki A , Arihiro K & Toge T (2006) Maturation of dendritic cells and T‐cell responses in sentinel lymph nodes from patients with breast carcinoma. Cancer 106, 1227–1236. 16475148 10.1002/cncr.21729

[19] Mansfield AS , Heikkila P , von Smitten K , Vakkila J & Leidenius M (2011) Metastasis to sentinel lymph nodes in breast cancer is associated with maturation arrest of dendritic cells and poor co‐localization of dendritic cells and CD8+ T cells. Virchows Arch 459, 391–398. 21894561 10.1007/s00428-011-1145-3

[20] Kohrt HE , Nouri N , Nowels K , Johnson D , Holmes S & Lee PP (2005) Profile of immune cells in axillary lymph nodes predicts disease‐free survival in breast cancer. PLoS Medicine 2, e284. 16124834 10.1371/journal.pmed.0020284 PMC1198041

[21] van Pul KM , Vuylsteke RJCLM , van de Ven R , te Velde EA , Rutgers EJT , van den Tol PM , Stockmann HBAC & de Gruijl TD (2019) Selectively hampered activation of lymph node‐resident dendritic cells precedes profound T cell suppression and metastatic spread in the breast cancer sentinel lymph node. J Immunother Cancer 7, 133. 10.1186/s40425-019-0605-1 31118093 PMC6530094

[22] Rakha EA , Starczynski J , Lee AHS & Ellis IO (2014) The updated ASCO/CAP guideline recommendations for HER2 testing in the management of invasive breast cancer: a critical review of their implications for routine practice. Histopathology 64, 609–615. 24382093 10.1111/his.12357

[23] Josefsson SE , Huse K , Kolstad A , Beiske K , Pende D , Steen CB , Inderberg EM , Lingjærde OC , Østenstad B , Smeland EB *et al*. (2018) T cells expressing checkpoint receptor TIGIT are enriched in follicular lymphoma tumors and characterized by reversible suppression of T‐cell receptor signaling. Clin Cancer Res 24, 870–881. 29217528 10.1158/1078-0432.CCR-17-2337 PMC5815910

[24] Blaker YN , Brodtkorb M , Maddison J , Hveem TS , Nesheim JA , Mohn HM , Kolstad A , Geisler CH , Liestøl K , Smeland EB *et al*. (2015) Computerized image analysis of the Ki‐67 proliferation index in mantle cell lymphoma. Histopathology 67, 62–69. 25431344 10.1111/his.12624

[25] Team R core (2015) R: A Language and Environment for Statistical Computing. R Foundation for Statistical Comping, Vienna, Austria.

[26] GraphPad (2014) GraphPad. LaJolla, CA. Available from: www.graphpad.com

[27] Klein CA (2013) Selection and adaptation during metastatic cancer progression. Nature 501, 365–372. 24048069 10.1038/nature12628

[28] Park D , Kåresen R , Naume B , Synnestvedt M , Beraki E & Sauer T (2009) The prognostic impact of occult nodal metastasis in early breast carcinoma. Breast Cancer Res Treat 118, 57–66. 19219629 10.1007/s10549-009-0340-2

[29] Bruggner RV , Bodenmiller B , Dill DL , Tibshirani RJ & Nolan GP (2014) Automated identification of stratifying signatures in cellular subpopulations. Proc Natl Acad Sci USA 111, E2770–E2777. 24979804 10.1073/pnas.1408792111 PMC4084463

[30] Joller N , Lozano E , Burkett PR , Patel B , Xiao S , Zhu C , Xia J , Tan TG , Sefik E , Yajnik V *et al*. (2014) Treg cells expressing the coinhibitory molecule TIGIT selectively inhibit proinflammatory Th1 and Th17 cell responses. Immunity 40, 569–581. 24745333 10.1016/j.immuni.2014.02.012 PMC4070748

[31] Sakaguchi S , Mikami N , Wing JB , Tanaka A , Ichiyama K & Ohkura N (2020) Regulatory T cells and human disease. Annu Rev Immunol 38, 541–566. 32017635 10.1146/annurev-immunol-042718-041717

[32] Balsat C , Blacher S , Herfs M , Van de Velde M , Signolle N , Sauthier P , Pottier C , Gofflot S , De Cuypere M , Delvenne P *et al*. (2017) A specific immune and lymphatic profile characterizes the pre‐metastatic state of the sentinel lymph node in patients with early cervical cancer. OncoImmunology 6, 1–10. 10.1080/2162402X.2016.1265718 PMC5353944 28344873

[33] Commerford CD , Dieterich LC , He Y , Hell T , Montoya‐Zegarra JA , Noerrelykke SF , Russo E , Röcken M & Detmar M (2018) Mechanisms of tumor‐induced lymphovascular niche formation in draining lymph nodes. Cell Rep 25, 3554–3563.e4. 30590031 10.1016/j.celrep.2018.12.002 PMC6315107

[34] Jackson HW , Fischer JR , Zanotelli VRT , Ali HR , Mechera R , Soysal SD , Moch H , Muenst S , Varga Z , Weber WP *et al*. (2020) The single‐cell pathology landscape of breast cancer. Nature 578, 615–620. 31959985 10.1038/s41586-019-1876-x

[35] Waickman AT & Powell JD (2012) mTOR, metabolism, and the regulation of T‐cell differentiation and function. Immunol Rev 249, 43–58. 22889214 10.1111/j.1600-065X.2012.01152.x PMC3419491

[36] Josefsson SE , Beiske K , Blaker YN , Førsund MS , Holte H , Østenstad B , Kimby E , Köksal H , Wälchli S , Bai B *et al*. (2019) TIGIT and PD‐1 mark intratumoral T cells with reduced effector function in B‐cell non‐hodgkin lymphoma. Cancer Immunol Res 7, 355–362. 30659053 10.1158/2326-6066.CIR-18-0351 PMC6636339

[37] Joller N , Hafler JP , Brynedal B , Kassam N , Spoerl S , Levin SD , Sharpe AH & Kuchroo VK (2011) Cutting edge: TIGIT has T cell‐intrinsic inhibitory functions. J Immunol 186, 1338–1342. 21199897 10.4049/jimmunol.1003081 PMC3128994

[38] Sato E , Olson SH , Ahn J , Bundy B , Nishikawa H , Qian F , Jungbluth AA , Frosina D , Gnjatic S , Ambrosone C *et al*. (2005) Intraepithelial CD8+ tumor‐infiltrating lymphocytes and a high CD8+/regulatory T cell ratio are associated with favorable prognosis in ovarian cancer. Proc Natl Acad Sci USA 102, 18538–18543. 16344461 10.1073/pnas.0509182102 PMC1311741

[39] Bates GJ , Fox SB , Han C , Leek RD , Garcia JF , Harris AL & Banham AH (2006) Quantification of regulatory T cells enables the identification of high‐risk breast cancer patients and those at risk of late relapse. J Clin Oncol 24, 5373–5380. 17135638 10.1200/JCO.2006.05.9584

[40] Lucca LE & Dominguez‐Villar M (2020) Modulation of regulatory T cell function and stability by co‐inhibitory receptors. Nat Rev Immunol 20, 680–693. 32269380 10.1038/s41577-020-0296-3

[41] De Simone M , Arrigoni A , Rossetti G , Gruarin P , Ranzani V , Politano C , Bonnal RJP , Provasi E , Sarnicola ML , Panzeri I *et al*. (2016) Transcriptional landscape of human tissue lymphocytes unveils uniqueness of tumor‐infiltrating T regulatory cells. Immunity 45, 1135–1147. 27851914 10.1016/j.immuni.2016.10.021 PMC5119953

[42] Núñez NG , Tosello Boari J , Ramos RN , Richer W , Cagnard N , Anderfuhren CD , Niborski LL , Bigot J , Meseure D , De La Rochere P *et al*. (2020) Tumor invasion in draining lymph nodes is associated with Treg accumulation in breast cancer patients. Nat Commun 11, 1–15. 32601304 10.1038/s41467-020-17046-2 PMC7324591

[43] Wang L , Simons DL , Lu X , Tu TY , Solomon S , Wang R , Rosario A , Avalos C , Schmolze D , Yim J *et al*. (2019) Connecting blood and intratumoral Treg cell activity in predicting future relapse in breast cancer. Nat Immunol 20, 1220–1230. 31285626 10.1038/s41590-019-0429-7 PMC8802768

[44] Dougall WC , Kurtulus S , Smyth MJ & Anderson AC (2017) TIGIT and CD96: new checkpoint receptor targets for cancer immunotherapy. Immunol Rev 276, 112–120. 28258695 10.1111/imr.12518

[45] Johnston RJ , Comps‐Agrar L , Hackney J , Yu X , Huseni M , Yang Y , Park S , Javinal V , Chiu H , Irving B *et al*. (2014) The immunoreceptor TIGIT regulates antitumor and antiviral CD8 + T cell effector function. Cancer Cell 26, 923–937. 25465800 10.1016/j.ccell.2014.10.018

[46] Rodriguez‐Abreu D , Johnson ML , Hussein MA , Cobo M , Patel AJ , Secen NM , Lee KH , Massuti B , Hiret S , Yang JC‐H *et al*. (2020) Primary analysis of a randomized, double‐blind, phase II study of the anti‐TIGIT antibody tiragolumab (tira) plus atezolizumab (atezo) versus placebo plus atezo as first‐line (1L) treatment in patients with PD‐L1‐selected NSCLC (CITYSCAPE). J Clin Oncol 38(15_suppl), 9503.

[47] Kamada T , Togashi Y , Tay C , Ha D , Sasaki A , Nakamura Y , Sato E , Fukuoka S , Tada Y , Tanaka A *et al*. (2019) PD‐1+ regulatory T cells amplified by PD‐1 blockade promote hyperprogression of cancer. Proc Natl Acad Sci USA 116, 9999–10008. 31028147 10.1073/pnas.1822001116 PMC6525547

