# Supplementary material for: Breast cancer metastasis: immune profiling of lymph nodes reveals exhaustion of effector T cells and immunosuppression
Source: Mol Oncol. 2021 Jul 12;16(1):88–103. doi: 10.1002/1878-0261.13047 (PMC8732351; doi:10.1002/1878-0261.13047)
Supplement: Supplementary file 1 — Fig. S1. Consistency of staining. Fig. S2. Mass cytometry antibody panel. Fig. S3. Gating scheme. Fig. S4. Citrus analysis on T cells. Fig. S5. Expression of TIGIT and TIGIT related proteins. Table S1. Clinical and histopathological parameters. Table S2. Mass cytometry antibody panel. Table S3. Antibodies used for Flow Cytometry analysis and TCR activation. Table S4. Comparison of CD4 and CD8 quantification in four patient samples (Fig. 5). Table S5. p‐values for abundance analysis (Fig. 2). Table S6. p‐values for marker expression analysis (Fig. 3). [file MOL2-16-88-s001.pdf]

Breast cancer metastasis: immune profiling of lymph nodes reveals exhaustion of effector T cells and immunosuppression

Inga Hansine Rye[1#], Kanutte Huse[2,3#], Sarah E. Josefsson[2,3], Wanja Kildal[4], Håvard E. Danielsen[4,5,6], Ellen Schlichting[7], Øystein Garred[8], Margit L. Riis[7], OSBREAC, Ole Christian Lingjærde[9], June H. Myklebust[2,3*] and Hege G. Russnes[1,8*]

Supplementary Material

Supplementary Figure 1

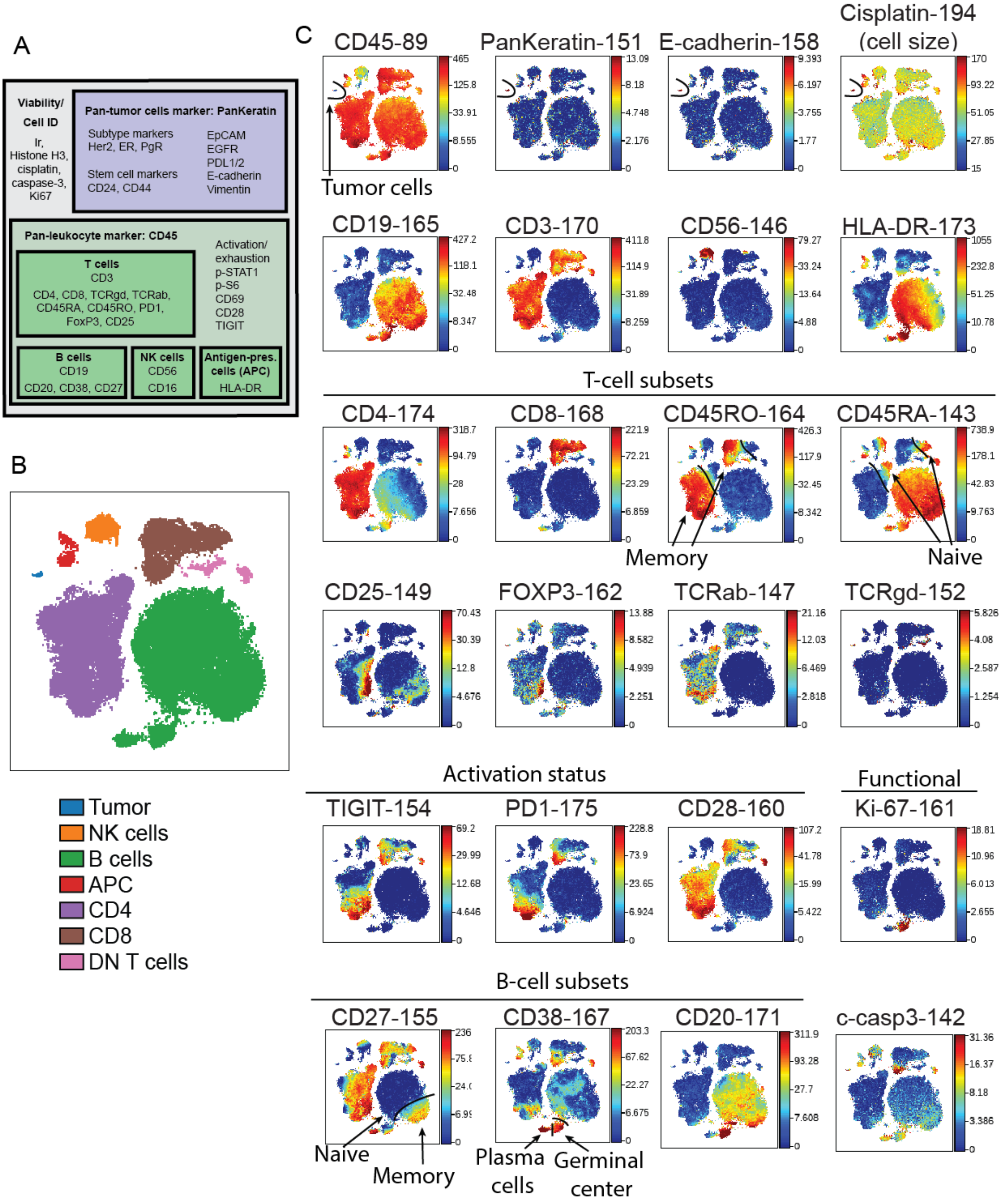

Supplementary Figure 1

A, Antibodies used to identify B-, T- and NK (natural killer)- cell lineages, antigen-presenting cells (APC), and tumor cells. **B-C**, viSNE map of all lineage markers on one representative sentinel lymph node positive for tumor cell. Each dot in the viSNE map represents a single cell and the position of each cell is based on similarity of the markers included in the viSNE analysis meaning that similar cells are positioned closed together in “islands”. **B**, Position of the populations in the viSNE map is

indicated by color coding. **C**, The color of the dot represents expression intensity of markers (target name followed by mass number of metal tag) as indicated. The black lines in the viSNE maps are drawn to visualize the population of interest indicated with arrows. PanKeratin and CD45 have a mutually exclusive staining pattern. The tumor cell marker E-cadherin (Ecad) is specific for the tumor cell island in the viSNE map. CD19, CD3, CD56 and HLA-DR were used to identify the four main immune cell populations; B cells, T cells, natural killer cells (NK) and non-B-cell antigen-presenting cells (APC). Cisplatin, primarily used as a live/dead cell marker, is also a marker for cell size and as expected, tumor cells were larger than the immune cells and antigen-presenting cells were larger than B, T and NK cells. Markers for subsets of B and T cells were specific for their expected populations. The proliferation marker Ki-67 is specific for the highly proliferative germinal center B cells and CD28 is positive on activated T cells.

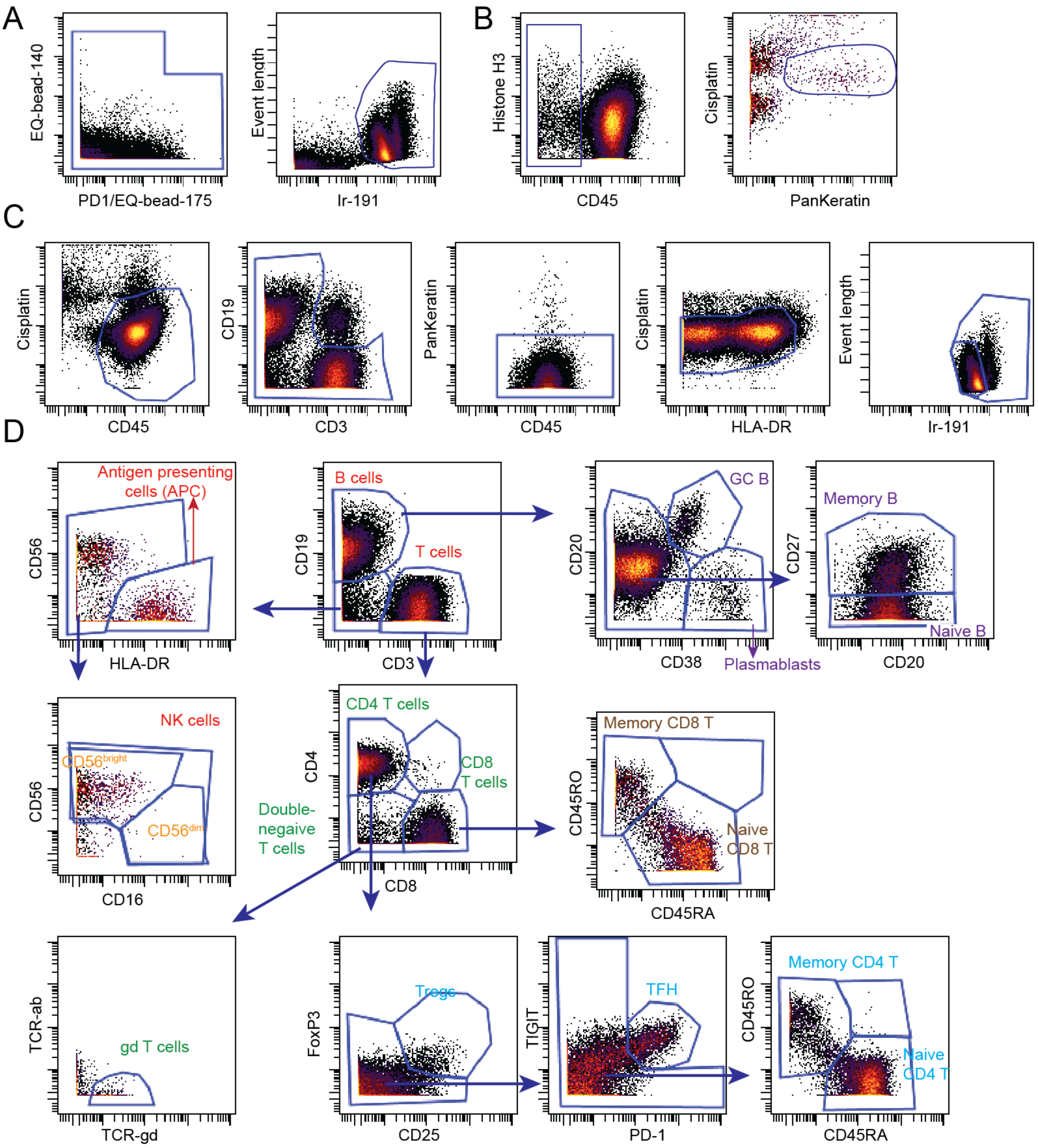

Supplementary Figure 2. Gating scheme

A, Pregating common to all populations. Exclude beads and exclude debris (all Ir-positive cells) **B**, Gating of tumor cells. CD45- cells, and PanKer+Cisplatin intermediate. **C**, Pregating for immune cells. Exclude CD45- and high cisplatin. Exclude T/B cell doublets. Exclude doublets with tumor cells. Tight live cell gate (not excluding larger APC). Tight singlet gate in Ir and event length. **D**, Gating of immune cell subsets.

Supplementary Figure 3

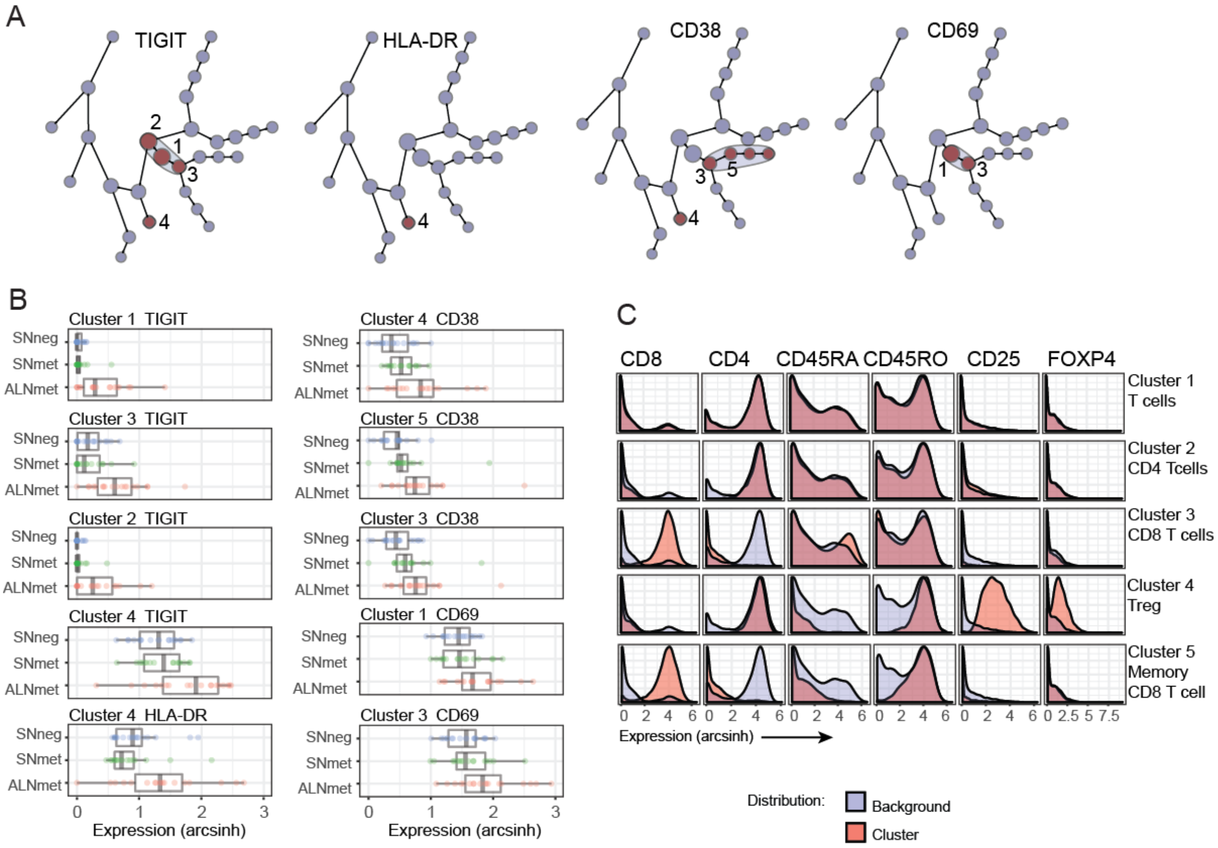

Supplementary Figure 3: CITRUS analysis of T cells

CITRUS analysis on T cells from all samples to identify differences in protein expression between SNneg (non metastatic sentinel node), SNmet (metastatic sentinel node) and ALNmet (metastatic axillary lymph node).

A, Feature plots showing clusters identified to differentially express markers as indicated. **B**, Expression of makers used for clustering in CITRUS analysis. Blue histograms represent the total T cell population and red histograms represent marker expression in each cluster. Manually labeled cell types based on phenotype are indicated for each cluster. **C**, Intensity of markers tested in each CITRUS cluster.

Supplementary Figure 4

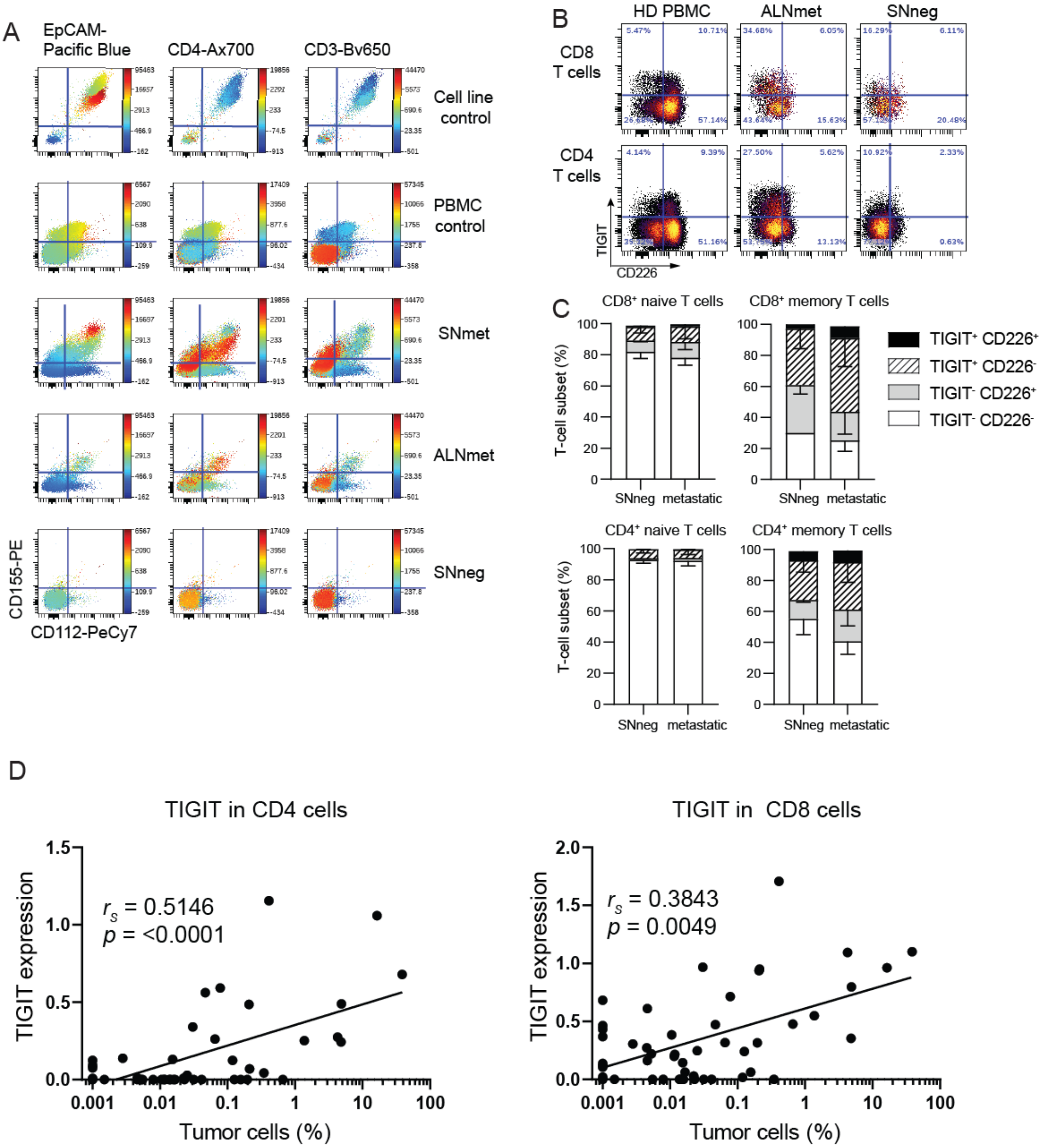

Supplementary Figure 4: Expression of TIGIT and TIGIT-related proteins
A, The same data as in Fig. 3C is presented. Expression of EpCAM, CD3 and CD4 indicate that both breast cancer cells and monocytes/macrophages express TIGIT ligands CD155 and CD112. **B-C**, The same data as in Fig. 3B is here presented separately for naive and memory T cells and also including the activating co-receptor CD226 which compete with TIGIT for binding to the same ligands. **B**, One representative healthy donor, ALNmet (metastatic axillary lymph node) and SNneg (non metastatic sentinel node). **C**, Mean values and SD is shown, *n* = 3 of each sample type. **D**, Correlation analysis of TIGIT expression vs. tumor percentage

measured by mass cytometry. The straight line was found by linear regression of TIGIT expression on the logarithm of tumor abundance. r_S = Spearman correlation coefficient

Supplemetary Figure 5

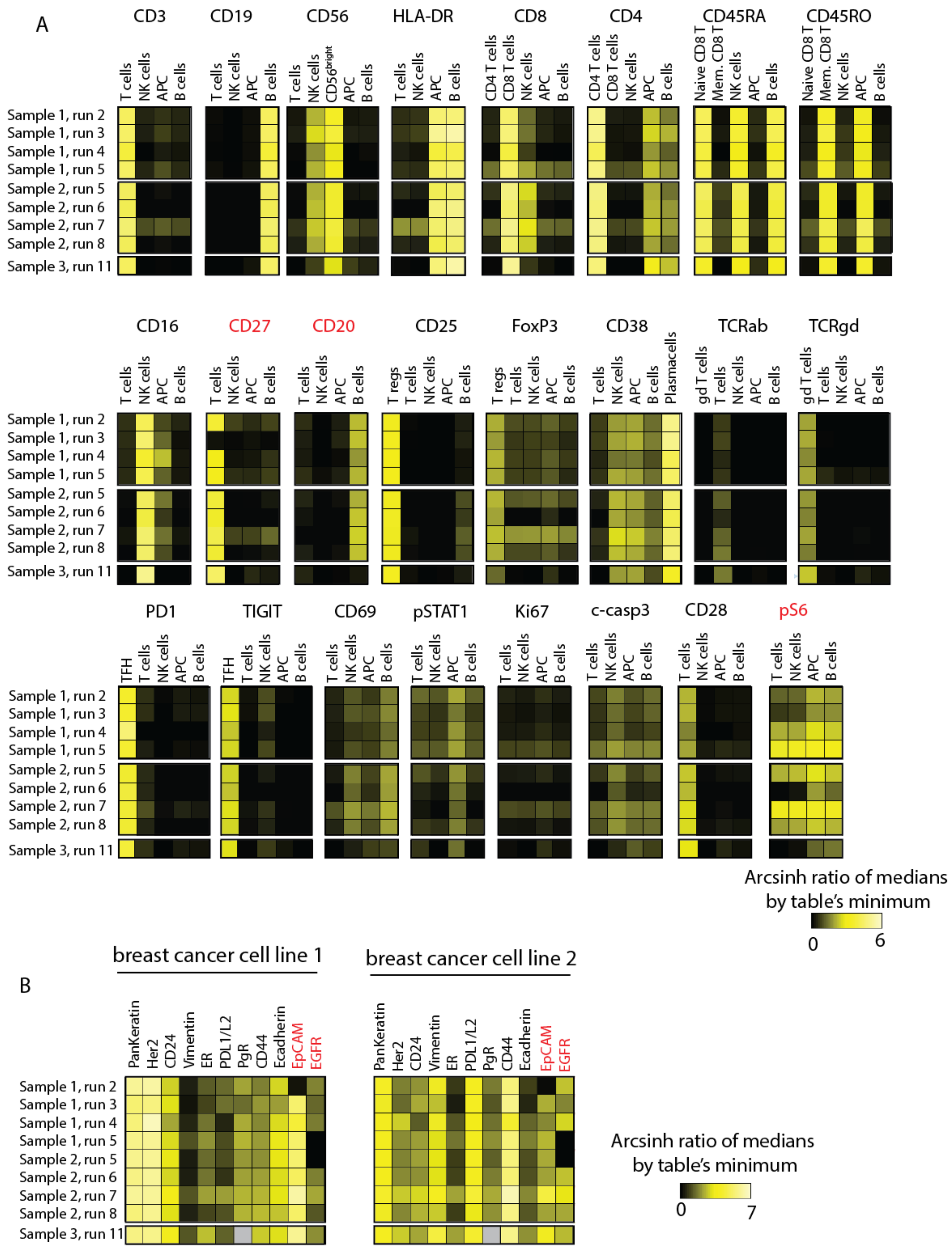

Supplementary Figure 5. Consistency of staining

A, Control for consistency in staining for immune cell markers in populations from PBMC included in control sample. Red text denotes markers where the variance across all controls was >0.5. Due to poor CD27 staining in run 3 and CD20 in run 11, these markers were not analyzed in samples from those runs, respectively. Variable pS6 staining was observed in several runs and pS6 was therefore excluded from all analyses. Scale is normalized to lowest level in each heatmap. **B,** Consistency of staining for tumor cell markers in breast cancer cell lines included in control sample. EpCAM and EGFR staining were variable and those markers were excluded from all analyses.

Supplementary Table 1: Clinical and histopathological parameters

PT = primary tumor; ER% = percentage positive cells for estrogen receptor; PgR% = percentage positive cells for progesterone receptor; HER2 = clinical HER2 score: 0=neg, 1=negative (weak), 3=positive; pN = Lymph node grading; #pos LN = number of metastatic LN (SN+ALN), # LN tot = total number of LN (SN+ALN) analyzed, NA = not available, ITC=isolated tumor cells.

Patient ID	Sample type	Subtype	PT Grade	PT ER%	PT PgR%	PT HER2	PT Ki67%	PT size(mm)	pN	# pos LN	# LN tot	SNmet size (mm)	ALNmet size (mm)
1	ALNmet	HER2	3	100	15	3	53	7	pN1	3	15	NA	NA
2	ALNmet	ER	3	100	100	0	65	40	pN2	5	16	NA	23
3	ALNmet	HER2	3	100	0	3	50	17	pN1	1	15	NA	25
4	SNneg	ER	2	100	100	0	26	11	pN0	0	2		
5	SNneg	HER2	3	0	0	3	75	35	pN0	0	2		
6	SNneg	TN	3	0	0	0	38	16	pN0	0	1		
7	SNneg	HER2	2	75	0	3	26	20	pN0	0	2		
8	ALNmet	TN	3	0	0	0	75	48	pN1	1	12	NA	13
9	ALNmet	HER2	NA	50	0	3	50	35	pN1	2	10	NA	25
10	SNneg	HER2	3	100	0	3	40	21	pN0	0	1		
11	SNneg	HER2	3	0	0	3	60	25	pN0	0	1		
12	SNmet	TN	3	0	0	0	90	21	pN1	3	18	18	
13	ALNmet	ER	3	80	0	3	80	17	pN2	7	11	NA	30
14	ALNmet	TN	3	0	0	0	80	21	pN3	12	18	NA	22
15	SNneg	TN	3	0	0	3	41	16	pN0	0	3		
16	SNneg	ER	2	90	40	1	50	18	pN0	0	1		
17	SNmet	ER	1	100	90	0	16	11	pN1	1	7	9	
18	ALNmet	TN	3	0	0	0	61	35	pN1	2	25	NA	15
19	ALNmet	TN	3	0	0	0	70	60	pN1	1	14	NA	15
20	ALNmet	TN	3	0	0	0	50	18	pN3	14	19	NA	9
21	SNmet	ER	1	100	100	0	15	16	pN1	2	10	4	
22	SNmet	HER2	3	50	0	3	47	22	pN1	1	2	5	
23	SNmet	ER	2	80	100	0	47	30	pN2	6	18	10	2.8
24	ALNmet	ER	2	50	50	1	29	50	pN1	2	14	21	14
25	ALNmet	HER2	3	0	0	3	70	40	pN1	1	8	NA	4
26	SNmet	TN	2	0	0	0	60	40	pN1	1	3	3	
27	SNmet	HER2	2	0	0	3	69	36	pN1	2	14	7	
28	SNneg	ER	2	100	100	1	10	18	pN0	0	2		
29	SNneg	TN	3	0	0	0	87	18	pN0	0	2		
30	SNneg	TN	3	0	0	0	95	12	pN0	0	3		
31	SNneg	ER	3	50	0	1	52	60	pN0	0	2		
32	SNneg	ER	3	20	0	0	75	23	pN0	0	1		
33	SNmet	HER2	3	0	0	3	70	13	pN1	2	4	11	

34	ALNmet	TN	3	0	0	0	80	15	pN2	6	11	NA	20
35	ALNmet	HER2	3	100	50	3	32	5	pN2	4	8	NA	33
36	SNmet	ER	2	100	100	0	33	20	pN1	1	1	8.5	
37	SNmet	ER	2	50	50	1	56	16	pN0(mi)	3	3	1.8	
38	SNmet	ER	2	80	90	0	20	35	pN0(i+)	1	2	ITC	
39	SNmet	ER	3	100	20	0	60	22	pN1	1	1	3.5	
40	ALNmet	ER	2	100	100	0	45	15	pN3	11	32	NA	12
41	SNmet	ER	2	50	50	0	25	16	pN1	1	11	10	
42	SNmet	ER	2	50	50	0	7	30	pN0(i+)	1	1	ITC	
43	SNmet	ER	2	90	0	0	41	30	pN0(mi)	1	1	0.3	
44	SNneg	ER	2	90	80	1	25	60	pN0	0	2		
45	SNneg	ER	3	100	80	1	24	24	pN0	0	3		
46	ALNmet	ER	2	100	10	0	50	30	pN1	1	13	NA	25
47	ALNmet	ER	3	100	0	1	41	28	pN1	3	15	NA	12
48	ALNmet	ER	3	90	60	1	37	25	pN1	1	14	NA	14
49	SNmet	TN	3	0	0	0	55	15	pN0(mi)	1	2	1.4	
50	SNneg	TN	3	0	0	0	90	16	pN0	0	1		
51	SNneg	TN	3	0	0	0	45	12	pN0	0	3		
52	SNneg	TN	1	0	0	0	13	6	pN0	0	2		

Supplementary Table 2: *p*-values for analyses in Figure 2 (abundance)

			Dunn's multiple comparisons test		
	Nominal *p*-value	Bonferroni-corrected *p*-value	SNneg vs. SNmet	SNneg vs. ALNmet	SNmet vs. ALNmet
APC	0.0240	n.s.			
NK cells	n.s.	n.s.			
CD56 bright	n.s.	n.s.			
CD56 dim	n.s.	n.s.			
B cells	n.s.	n.s.			
Memory B cells	n.s.	n.s.			
naive B cells	n.s.	n.s.			
GC B cells	n.s.	n.s.			
Plasma cells	0.0457	n.s.			
T cells	n.s.	n.s.			
CD4 T cells	<0.0001	<0.0023	n.s.	<0.0001	0.0023
CD8 T cells	<0.0001	<0.0023	n.s.	<0.0001	0.0093
DP T cells	n.s.	n.s.			
gd T cells	0.0008	0.0184	n.s.	0.0033	0.0033
Tregs	0.0038	n.s.			
TFH	0.0041	n.s.			
Naive CD4	0.0001	0.0023	n.s.	0.0005	0.0015
Memory CD4	0.0004	0.0092	n.s.	0.0011	0.0040
RARO CD4	0.0007	0.0161	0.0351	n.s.	0.0005

Naive CD8	0.0006	0.0138	n.s.	0.0466	0.0004
Memory CD8	0.0006	0.0138	n.s.	0.0439	0.0005
RARO CD8	0.0028	n.s.			
DN T cells	0.0010	0.023	n.s	0.0009	0.0360

Supplementary Table 3. *p*-values for analyses in Figure 3 (marker expression)

				Dunn's multiple comparisons test		
Marker	Populations	Nominal p-value	Corrected p-value	SNneg vs. SNmet	SNneg vs. ALNmet	SNmet vs. ALNmet
TIGIT	T cells	<0.0001	<0.001	n.s.	0.0001	0.0026
TIGIT	CD4 T cells	0.001	0.01	n.s.	0.0011	0.0271
TIGIT	CD8 T cells	0.0003	0.003	n.s.	0.0031	0.0007
TIGIT	Tregs	0.0016	0.016	n.s.	0.0029	0.013
CD38	CD8 T cells	0.0066	n.s.			
CD38	Memory CD8 T cells	0.0019	0.019	n.s.	0.0012	n.s.
CD38	Tregs	0.0045	0.045	n.s.	0.0098	0.02
HLA-DR	Tregs	0.0004	0.004	n.s.	0.0069	0.0007
CD69	T cells	0.0399	n.s.			
CD69	CD8 T cells	n.s.				

Supplementary Table 4: Mass cytometry antibody panel

Tag	Target	Clone	vendor	Catalog #
89Y	CD45	HI30	Fluidigm	3089003B
141Pr	EpCAM (CD326)	9C4	Fluidigm	3141006B
142Nd	Caspase 3 (Cleaved)	D3E9	Fluidigm	3142004A
143Nd	CD45RA	HI100	Fluidigm	3143006B
144Nd	CD69	FN50	Fluidigm	3144018B
145Nd	PgR	D8Q2J	Fluidigm	3145011A
146Nd	CD56	HCD56	BioLegend	318345
147Sm	TCRab	IP26	BioLegend	306702
148Nd	HER2 (CD340)	29D8	Fluidigm	3148011A
149Sm	CD25 (IL-2R)	2A3	Fluidigm	3149010B
150Nd	CD44	IM7	Fluidigm	3150018B
151Eu	PanKer	C11	ThermoFisher	MA1-12594
152Sm	TCRgd	11F2	Fluidigm	3152008B
153Eu	pStat1 [Y701]	58D6	Fluidigm	3153003A
154Sm	TIGIT	MBSA43	Fluidigm	3154016B
155Gd	CD27	L128	Fluidigm	3155001B
156Gd	vimentin	RV202	Fluidigm	3156023A
158Gd	Ecad (CD324)	24E10	Fluidigm	3158021A
159Tb	PDL1	29E.2A3	Fluidigm	3159029B
159Tb	PDL2	24F.10C12	BioLegend	329613
160Gd	CD28	CD28.2	Fluidigm	3160003B
161Dy	Ki-67	B56	Fluidigm	3162012B
162Dy	Foxp3	PCH101	Fluidigm	3162011A

163Dy	ER	D8H8	Fluidigm	3163024A
164Dy	CD45RO	UCHL1	Fluidigm	3164007B
165Ho	CD19	HIB19	Fluidigm	3165025B
166Er	CD24	ML5	Fluidigm	3166007B
167Er	CD38	HIT2	Fluidigm	3167001B
168Er	CD8a	SK1	Fluidigm	3168002B
169Gd	EGFR	D38B1	Fluidigm	3169017A
170Er	CD3	UCHT1	Fluidigm	3170001B
171Yb	CD20	2H7	Fluidigm	3171012B
172Yb	pS6 [S235/S236]	N7-548	Fluidigm	3172008A
173Yb	HLA-DR	L243	Fluidigm	3173005B
174Yb	CD4	SK3	Fluidigm	3174004B
175Lu	CD279 (PD-1)	EH12.2H7	Fluidigm	3175008B
176Yb	Histone3	D1H2	Fluidigm	3176016A
191/193Ir	Cell-ID Intercalator Ir		Fluidigm	201192B
194Pt	Cell-ID Cisplatin-194Pt		Fluidigm	201194
209Bi	CD16	3G8	Fluidigm	3209002B

Supplementary Table 5: Antibodies used for flow cytometry analysis and TCR activation

	Ab	Fluorophore	Catalog	Residue	Clone	Vendor	Dilution
Phospho-specific antibodies	p-ERK 1/2	PE	14095S	T202/Y204	197G2	Cell Signaling	1:50
	p-CD3ζ	PE	558448	Y142	K25-407.69	BD	1:10
	p-SLP76	Alexa Fluor 488	558439	Y128	J141-668.36.58	BD	1:10
	p-S6	Alexa Fluor 488	4851S	S235/236	D57.2.2E	Cell Signaling	1:50
Lineage markers, used with phospho-specific antibodies and in immunophenotyping	CD45RO	BV510	304246		UCHL1	Biolegend	1:50
	CD8	BV785	301046		RPA-T8	Biolegend	1:40
	CD4	PerCPCy5.5	300530		RPA-T4	Biolegend	1:20
	CD4	Alexa Fluor 700	300526		RPA-T4	Biolegend	1:100
	CD38	PeCy7	356608		HB-7	Biolegend	1:50
	TIGIT	APC	17-9500-42		MBSA43	eBioscience	1:20
	CD3	BV650	300468		UCHT1	Biolegend	1:50
	EpCAM	Pacific Blue	324217		9C4	Biolegend	1:20
	CD155	PE	337610		SKII.4	Biolegend	1:20
	CD112	PeCy7	337414		TX31	Biolegend	1:40
	CD226	PerCPCy5.5	337314		11A8	Biolegend	1:40
Antibodies used for TCR activation (α-TCR)	CD3 Biotin		13-0037-82		OKT-3	eBioscience	
	CD28 Biotin		13-0289-82		CD28.2	eBioscience	

SupplementaryTable 6. Comparison of CD4 and CD8 quantification in 4 patient samples, illustrated in Figure 4

Percentage of CD4 and CD8 positive cells of total CD45 found by mass cytometry

and the objective IHC scoring by Immunopath (fieldfraction, measuring percentage of positive pixels in IHC images) of CD4, CD8 and AE1/AE3 both from the tumor area (identified as AE1/AE3 positive areas) and the whole tissue section (Fig. 4).

	**Mass cytometry (% of CD45)**		**ImmunoPath (% positive area)**		**Overlap of scoring (%)**	
**ID**	CD4	CD8	CD4	CD8	CD4	CD8
Patient 11, SNneg	68.2	10.2	68.6	14.3	99	71
Patient 27, SNmet	45.5	20.1	51.4	24.8	89	81
Patient 37, SNmet	49.6	13.6	87.6	23.1	57	59
Patient 39, SNmet	61.3	11	87.7	21.4	70	51
